# Crystal structure of the formal 20 electron zirconocene penta­fulvene complex Cp_2_Zr(η^5^,η^1^-adamantyl­idene­penta­fulvene):toluene:*n*-hexane = 1:0.125:0.125

**DOI:** 10.1107/S2056989017015560

**Published:** 2017-11-03

**Authors:** Malte Fischer, Marc Schmidtmann, Rüdiger Beckhaus

**Affiliations:** a Institut für Chemie, Fakultät für Mathematik und Naturwissenschaften, Carl von Ossietzky Universität Oldenburg, 26129 Oldenburg, Germany

**Keywords:** crystal structure, zirconium, metallocene, zirconocene, penta­fulvene, 20 electron complex

## Abstract

The crystal structure of a solvated zirconocene penta­fulvene complex with a bulky adamantyl­idene substitution pattern, namely (η^5^,η^1^-adamantyl­idene­penta­fulvene)bis­(η^5^-cyclo­penta­dien­yl)zirconium(IV)–toluene–*n*-hexane (8/1/1), [Zr(C_15_H_18_)(C_5_H_5_)_2_]·0.125C_7_H_8_·0.125C_6_H_14_, is reported. Reducing zirconocene dichloride with magnesium results in the formation of a low-valent zirconocene reagent that reacts readily with adamantyl­idene­penta­fulvene to give the aforementioned complex. Single crystal X-ray diffraction proves the dianion-like η^5^:η^1^ binding mode of the fulvene ligand to the central Zr^IV^ atom. The asymmetric unit contains four independent mol­ecules of [η^5^:η^1^-adamantyl­idene­penta­fulvene]bis­[(η^5^)-cyclo­penta­dien­yl]zirconium(IV), together with half a mol­ecule of toluene disordered with half a mol­ecule of *n*-hexane (the solvent mol­ecules have no direct influence on the complex). In each of the four complex mol­ecules, the central Zr^IV^ atom has a distorted tetra­hedral coordination environment. The measured crystal consisted of two domains with a refined ratio of 0.77:0.23.

## Abstract 2

The mol­ecular and crystal structure of a formal 20 electron zirconium(IV) complex bearing two cyclo­penta­dienyl and one sterically demanding penta­fulvene ligand is reported in which the penta­fulvene is bound in an η^5^:η^1^ manner. The complex crystallizes together with toluene and *n*-hexane in a ratio of 1:0.125:0.125.

## Chemical context   

Over the last few decades, penta­fulvenes have found plenty of applications in organometallic chemistry (Preethalayam *et al.*, 2017 ▸; Neuenschwander, 1989 ▸), one of which is their use as versatile ligands for a variety of early and late transition metals featuring a multitude of coordination modes and reactivity patterns (Preethalayam *et al.*, 2017 ▸; Kreindlin & Rybinskaya, 2004 ▸). Whereas for late transition metals η^2^- and η^4^-bindng modes are known (Kim *et al.*, 2000 ▸; Rais & Bergman, 2004 ▸), most metals are bound in an η^6^-manner, either in a neutral olefinic η^2^:η^2^:η^2^ (Konietzny *et al.*, 2010 ▸) or in a dianionic η^5^:η^1^ fashion (Ebert *et al.*, 2014 ▸). The change of polarity at the exocyclic carbon atom of the penta­fulvene ligand, resulting from its bonding to the central metal atom, enables a multitude of insertion reactions and C—H-activation reactions that are of great inter­est to our research group (Ebert *et al.*, 2014 ▸; Manssen *et al.*, 2015 ▸, 2017 ▸; Oswald *et al.*, 2016 ▸) . In this context we have recently reported the syntheses of the first zirconocene-based penta­fulvene complexes and their reactivities (Jaroschik *et al.*, 2017 ▸). Here we report the synthesis and crystal structure of the solvated title compound, (η^5^,η^1^-adamantyl­idene­penta­fulvene)bis­(η^5^-cyclo­penta­dien­yl)zirconium(IV), **1**.
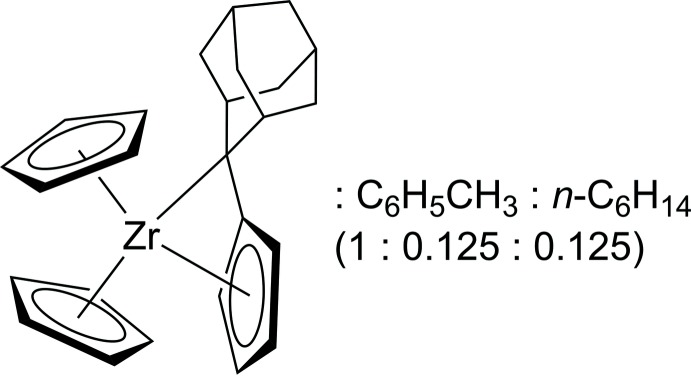


## Structural commentary   

**Figure 1:**
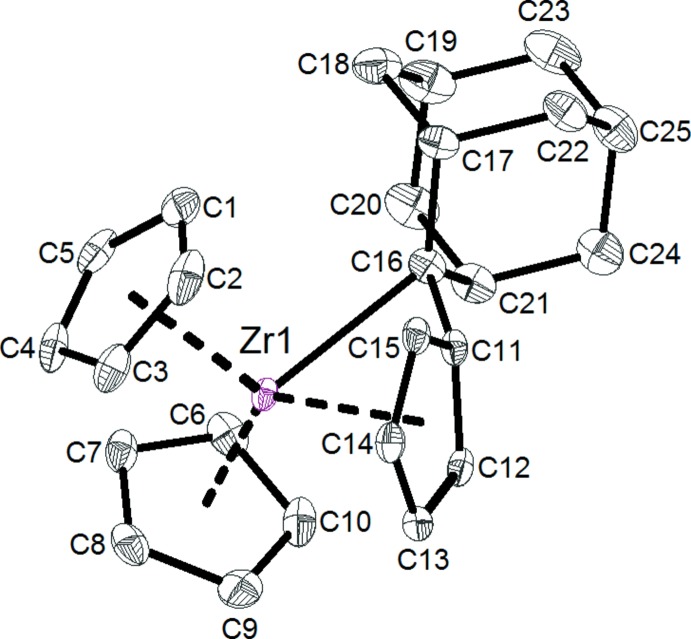
One of the four independent complex mol­ecules in the crystal structure of **1**. Displacement ellipsoids are drawn at the 50% probability level. H atoms and solvent mol­ecules have been omitted for clarity.

Compound **1** crystallizes in the triclinic space group *P* 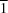 with four formula units per asymmetric unit together with one disordered solvent mol­ecule (ratio toluene:*n-*hexane = 1:1). Fig. 1 ▸ shows one of the complex mol­ecules present in the crystal of **1**. As a result of the high similarities with respect to structural parameters (bond lengths and angles) of the four complexes in the asymmetric unit, only this complex (Zr1) is discussed in detail. The mol­ecular structure shows the zirconium(IV) atom to be in a distorted tetra­hedral coordination environment. The zirconium atom lies 0.21 Å above the plane defined by the three centroids of the penta­fulvene and cyclo­penta­dienyl ligands, which is in good agreement with related complexes, *e.g*. 0.20 Å for the analogous complex with a 6,6′-di-*para*-tolyl­fulvene substitution pattern (Jaroschik *et al.*, 2017 ▸) and 0.20 Å for Cp_3_ZrH (Edelbach *et al.*, 1999 ▸). The mol­ecular structure of **1** in the solid state clearly confirms the π–η^5^:*σ–η* ^1^ binding mode of the fulvene moiety to the central metal atom. Characteristic parameters for this coordination mode are the deviation (bend angle *θ*) of the C_exo_—C_*ipso*_ bond toward the central zirconium(IV) atom (29.4°) as well as the ring slippage (Δ) toward the C_*ipso*_ atom of the five-membered ring of the penta­fulvene ligand (0.318 Å). The bond between the zirconium(IV) atom and the exocyclic carbon atom [Zr1—C16 = 2.605 (3) Å] is considerably longer than those of other zirconium complexes [Kraft *et al.*, 2002 ▸ (2.37 Å); Novarino *et al.*, 2011 ▸ (2.37 Å)], indicating a weak Zr—C_exo_ contact, but in good agreement with [π–η^5^:*σ–η* ^1^-C_5_H_4_=C(*para*-tol­yl)_2_]Zr(THF) (2.70 and 2.71 Å) reported previously by our group (Ebert *et al.*, 2014 ▸). Regarding the fulvene moiety, the coordination to the zirconocene fragment leads to the loss of the alternating single- and double-bond pattern of free penta­fulvene. This is indicated by the narrow range of the C—C bond lengths within the five-membered ring of the fulvene ligand [1.406 (4) to 1.437 (4) Å] in comparison with free fulvene [1.327 (3) to 1.459 (2) Å] (Garcia *et al.*, 1989 ▸). Hence, the hybridization of the exocyclic carbon atom lies between *sp* ^2^ and *sp* ^3^, which is further confirmed by the sum of angles around the C16 carbon atom [C11—C16—C17 = 116.9 (2)°, C17—C16—C21 = 109.4 (2)°, C11—C16—C21 = 118.7 (3)° = 345°].

## Supra­molecular features   

**Figure 2:**
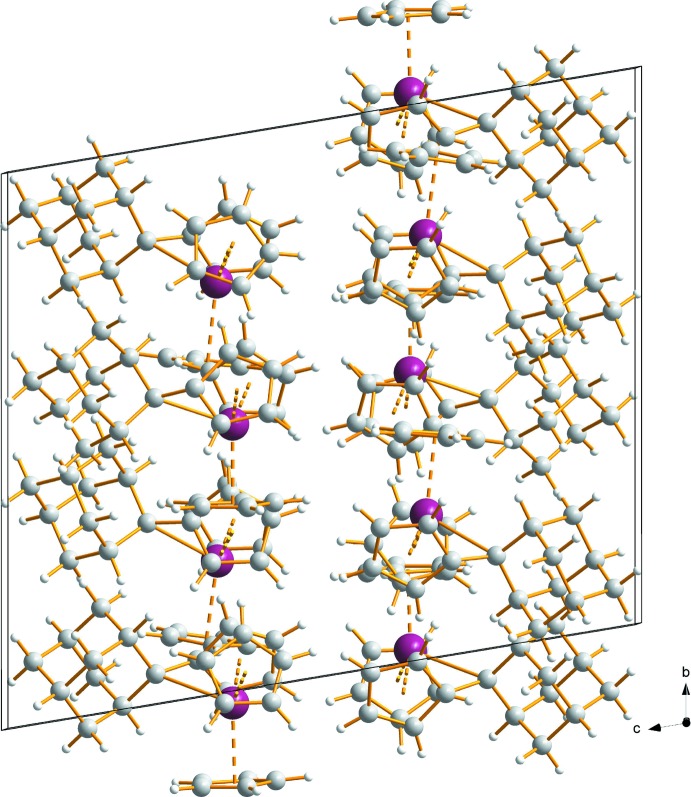
A view along the *a* axis showing the packing of mol­ecules in the crystal structure of compound **1**. Solvent mol­ecules have been omitted for clarity. No significant supra­molecular features can be observed. Color code: C grey, H white, Zr plum spheres.

**Figure 3:**
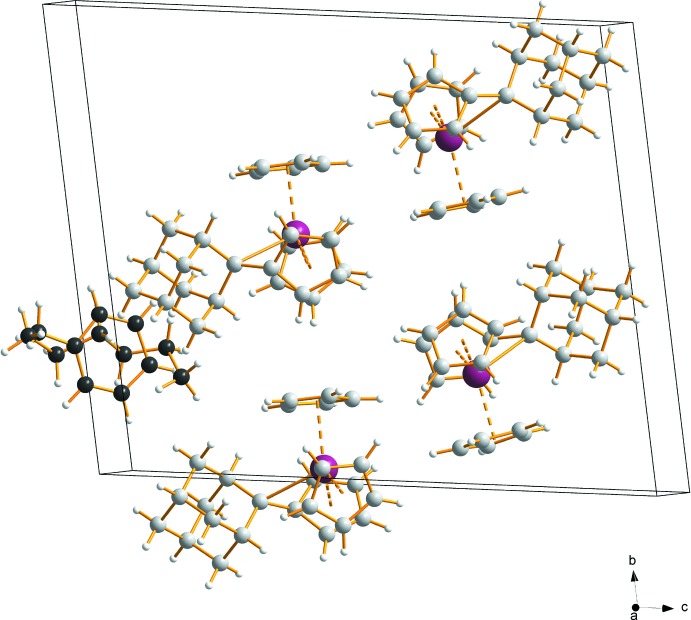
A view along the *a* axis showing the packing of mol­ecules in the asymmetric unit. Color code: C grey, H white, Zr plum spheres. Solvent mol­ecules are highlighted in black.

No significant supra­molecular features between the complex mol­ecules or between the complex mol­ecules and the solvent mol­ecules are observed. Hence the inter­molecular forces appear to be dominated by van der Waals inter­actions only. In the crystal structure of **1**, the solvent mol­ecules are located in the voids resulting from the packing arrangements of the complex mol­ecules. Fig. 2 ▸ shows the packing without solvent mol­ecules and Fig. 3 ▸ the packing with the contribution of the solvents.

## Synthesis and crystallization   

All reactions were carried out under a dry nitro­gen atmos­phere using Schlenk techniques or in a glove box. Zirconocene dichloride was purchased from Strem Chemicals and used as received. Adamantyl­idene­penta­fulvene was prepared according to a published procedure (Miller & Bercaw, 2006 ▸). Solvents were dried according to standard procedures over Na/K alloy with benzo­phenone as indicator and distilled under a nitro­gen atmosphere.

Zirconocene dichloride (1.000 g, 3.421 mmol), magnesium (0.083 g, 3.421 mmol) and adamantylidenefulvene (0.884 g, 3.421 mmol) were added to a Schlenk tube under argon. THF (40 ml) was added, and the reaction was stirred for 16 h at room temperature. THF was evaporated under vacuum and 40 ml of toluene were added to the crude product. After filtration, toluene was evaporated under vacuum to give **1** as a yellow solid in 81% yield.

Crystals suitable for single crystal X-ray diffraction were obtained from a saturated solution of **1** in toluene, layered with *n*-hexane at room temperature.

## Refinement   

Crystal data, data collection and structure refinement details are summarized in Table 1 ▸.

The measured crystal consisted of two domains. *TWINABS* was therefore used to model the absorption correction and to generate a reflection file in the HKLF5 format. The refined ratio of the two domains was 0.77:0.23. Hydrogen atoms bonded to the carbon atoms were located from difference-Fourier maps but were subsequently fixed to idealized positions using appropriate riding models with *U*_iso_(H) = 1.2*U*_eq_(C). Reflections (001) and (00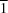) were obstructed from the primary beam stop and consequently omitted from the refinement. The solvent mol­ecules toluene and *n*-hexane were located from difference maps and refined with RIGU commands, with site occupancies fixed to 0.50 each.

## Supplementary Material

Crystal structure: contains datablock(s) I. DOI: 10.1107/S2056989017015560/wm5418sup1.cif

Structure factors: contains datablock(s) I. DOI: 10.1107/S2056989017015560/wm5418Isup2.hkl

Supporting information file. DOI: 10.1107/S2056989017015560/wm5418Isup3.cdx

CCDC reference: 1582067

Additional supporting information:  crystallographic information; 3D view; checkCIF report

## Figures and Tables

**Table 1:** Experimental details

Crystal data	
Chemical formula	[Zr(C_15_H_18_)(C_5_H_5_)_2_]·0.125C_7_H_8_·0.125C_6_H_14_
*M*_r_	441.98
Crystal system, space group	Triclinic, *P* 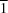
Temperature (K)	105
*a*, *b*, *c* (Å)	13.6751 (6), 16.0733 (7), 19.5889 (9)
α, β, γ (°)	98.6919 (18), 109.4236 (16), 90.5484 (16)
*V* (Å^3^)	4005.8 (3)
*Z*	8
Radiation type	Mo *K*α
μ (mm^−1^)	0.56
Crystal size (mm)	0.28 × 0.24 × 0.04

Data collection	
Diffractometer	Bruker APEXII CCD
Absorption correction	Multi-scan (*TWINABS*; Bruker, 2013 ▸)
*T*_min_, *T*_max_	0.900, 1.000
No. of measured, independent and observed [*I* > 2σ(*I*)] reflections	67120, 67120, 48102
(sin θ/λ)_max_ (Å^−1^)	0.746

Refinement	
*R*[*F* ^2^ > 2σ(*F* ^2^)], *wR*(*F* ^2^), *S*	0.044, 0.110, 1.01
No. of reflections	67120
No. of parameters	1058
No. of restraints	72
H-atom treatment	H-atom parameters constrained
Δρ_max_, Δρ_min_ (e Å^−3^)	1.04, −0.91

Computer programs: *APEX2* and *SAINT* (Bruker, 2013 ▸), *SHELXS2013* (Sheldrick, 2015*a* ▸), *SHELXL2014* (Sheldrick, 2015*b* ▸), *DIAMOND* (Brandenburg & Putz, 2006 ▸) and *publCIF* (Westrip, 2010 ▸).

## supplementary crystallographic information

### Crystal data

**Table:**

[Zr(C_15_H_18_)(C_5_H_5_)_2_]·0.125C_7_H_8_·0.125C_6_H_14_	*Z* = 8
*M**_r_* = 441.98	*F*(000) = 1844
Triclinic, *P*1	*D*_x_ = 1.466 Mg m^−^^3^
*a* = 13.6751 (6) Å	Mo *K*α radiation, λ = 0.71073 Å
*b* = 16.0733 (7) Å	Cell parameters from 6013 reflections
*c* = 19.5889 (9) Å	θ = 2.2–31.9°
α = 98.6919 (18)°	µ = 0.56 mm^−^^1^
β = 109.4236 (16)°	*T* = 105 K
γ = 90.5484 (16)°	Plate, yellow
*V* = 4005.8 (3) Å^3^	0.28 × 0.24 × 0.04 mm

### Data collection

**Table:**

Bruker APEXII CCD diffractometer	67120 independent reflections
Radiation source: sealed tube	48102 reflections with *I* > 2σ(*I*)
φ and ω scans	θ_max_ = 32.0°, θ_min_ = 1.3°
Absorption correction: multi-scan (*TWINABS*; Bruker, 2013)	*h* = −20→20
*T*_min_ = 0.900, *T*_max_ = 1.000	*k* = −23→23
67120 measured reflections	*l* = −29→29

### Refinement

**Table:**

Refinement on *F*^2^	Primary atom site location: structure-invariant direct methods
Least-squares matrix: full	Secondary atom site location: difference Fourier map
*R*[*F*^2^ > 2σ(*F*^2^)] = 0.044	Hydrogen site location: difference Fourier map
*wR*(*F*^2^) = 0.110	H-atom parameters constrained
*S* = 1.01	*w* = 1/[σ^2^(*F*_o_^2^) + (0.040*P*)^2^ + 4.*P*] where *P* = (*F*_o_^2^ + 2*F*_c_^2^)/3
67120 reflections	(Δ/σ)_max_ = 0.002
1058 parameters	Δρ_max_ = 1.04 e Å^−^^3^
72 restraints	Δρ_min_ = −0.91 e Å^−^^3^

### Special details

**Table:**

Geometry. All esds (except the esd in the dihedral angle between two l.s. planes) are estimated using the full covariance matrix. The cell esds are taken into account individually in the estimation of esds in distances, angles and torsion angles; correlations between esds in cell parameters are only used when they are defined by crystal symmetry. An approximate (isotropic) treatment of cell esds is used for estimating esds involving l.s. planes.
Refinement. Refined as a 2-component twin

### Fractional atomic coordinates and isotropic or equivalent isotropic displacement parameters (Å^2^)

**Table:**

	*x*	*y*	*z*	*U*_iso_*/*U*_eq_	Occ. (<1)
Zr1	0.34828 (2)	0.23916 (2)	0.66261 (2)	0.01264 (6)	
C1	0.4036 (2)	0.11710 (19)	0.7380 (2)	0.0277 (7)	
H1	0.4576	0.1313	0.7840	0.033*	
C2	0.4159 (2)	0.08779 (19)	0.6707 (2)	0.0295 (8)	
H2	0.4799	0.0757	0.6634	0.035*	
C3	0.3174 (2)	0.07918 (17)	0.61545 (19)	0.0239 (6)	
H3	0.3034	0.0645	0.5641	0.029*	
C4	0.2435 (2)	0.09648 (18)	0.65087 (19)	0.0236 (6)	
H4	0.1702	0.0916	0.6276	0.028*	
C5	0.2957 (2)	0.12171 (19)	0.72507 (19)	0.0260 (7)	
H5	0.2645	0.1391	0.7612	0.031*	
C6	0.2160 (2)	0.33703 (19)	0.69904 (18)	0.0222 (6)	
H6	0.2347	0.3591	0.7498	0.027*	
C7	0.15621 (19)	0.26119 (18)	0.66255 (18)	0.0211 (6)	
H7	0.1248	0.2244	0.6841	0.025*	
C8	0.1513 (2)	0.24974 (19)	0.58924 (18)	0.0232 (6)	
H8	0.1178	0.2029	0.5527	0.028*	
C9	0.2046 (2)	0.3198 (2)	0.57885 (19)	0.0258 (6)	
H9	0.2131	0.3286	0.5342	0.031*	
C10	0.2429 (2)	0.37398 (18)	0.6462 (2)	0.0251 (7)	
H10	0.2805	0.4267	0.6550	0.030*	
C11	0.50795 (18)	0.31699 (16)	0.70541 (16)	0.0149 (5)	
C12	0.45190 (19)	0.35608 (17)	0.64371 (16)	0.0162 (5)	
H12	0.4286	0.4116	0.6464	0.019*	
C13	0.43710 (19)	0.29892 (18)	0.57876 (16)	0.0175 (5)	
H13	0.3993	0.3081	0.5305	0.021*	
C14	0.48798 (19)	0.22570 (18)	0.59757 (16)	0.0175 (5)	
H14	0.4904	0.1769	0.5642	0.021*	
C15	0.53463 (18)	0.23734 (17)	0.67431 (16)	0.0158 (5)	
H15	0.5770	0.1987	0.7013	0.019*	
C16	0.48953 (19)	0.32835 (18)	0.77477 (16)	0.0175 (5)	
C17	0.5652 (2)	0.29025 (19)	0.83666 (16)	0.0202 (6)	
H17	0.5805	0.2326	0.8171	0.024*	
C18	0.5207 (2)	0.2850 (2)	0.89840 (18)	0.0282 (7)	
H18A	0.4553	0.2488	0.8790	0.034*	
H18B	0.5707	0.2595	0.9377	0.034*	
C19	0.4996 (2)	0.3735 (2)	0.92980 (19)	0.0327 (8)	
H19	0.4703	0.3698	0.9698	0.039*	
C20	0.4203 (2)	0.4103 (2)	0.86754 (18)	0.0304 (7)	
H20A	0.3550	0.3740	0.8482	0.036*	
H20B	0.4044	0.4672	0.8868	0.036*	
C21	0.4643 (2)	0.41597 (19)	0.80549 (17)	0.0225 (6)	
H21	0.4128	0.4405	0.7654	0.027*	
C22	0.6668 (2)	0.3476 (2)	0.86836 (18)	0.0243 (6)	
H22A	0.7178	0.3233	0.9080	0.029*	
H22B	0.6971	0.3510	0.8294	0.029*	
C23	0.5999 (3)	0.4300 (2)	0.96032 (19)	0.0333 (8)	
H23A	0.6512	0.4062	1.0003	0.040*	
H23B	0.5857	0.4869	0.9808	0.040*	
C24	0.5656 (2)	0.4735 (2)	0.83771 (19)	0.0281 (7)	
H24A	0.5949	0.4790	0.7986	0.034*	
H24B	0.5504	0.5305	0.8574	0.034*	
C25	0.6443 (2)	0.4363 (2)	0.89874 (19)	0.0286 (7)	
H25	0.7101	0.4731	0.9187	0.034*	
Zr2	0.33274 (2)	0.74120 (2)	0.66375 (2)	0.01197 (5)	
C26	0.4932 (2)	0.8472 (3)	0.6977 (2)	0.0361 (9)	
H26	0.5029	0.8891	0.7395	0.043*	
C27	0.4365 (2)	0.8540 (2)	0.6258 (2)	0.0276 (7)	
H27	0.4031	0.9024	0.6094	0.033*	
C28	0.4371 (2)	0.7770 (2)	0.58141 (18)	0.0273 (7)	
H28	0.4010	0.7625	0.5302	0.033*	
C29	0.5009 (3)	0.7261 (2)	0.6268 (2)	0.0380 (10)	
H29	0.5188	0.6713	0.6111	0.046*	
C30	0.5334 (2)	0.7678 (3)	0.6977 (2)	0.0423 (10)	
H30	0.5760	0.7464	0.7396	0.051*	
C31	0.3605 (3)	0.6196 (2)	0.7418 (2)	0.0377 (8)	
H31	0.3838	0.6372	0.7934	0.045*	
C32	0.4224 (3)	0.6016 (2)	0.6991 (3)	0.0407 (10)	
H32	0.4961	0.6020	0.7167	0.049*	
C33	0.3600 (4)	0.5831 (2)	0.6271 (3)	0.0476 (12)	
H33	0.3828	0.5697	0.5861	0.057*	
C34	0.2572 (3)	0.5873 (2)	0.6246 (3)	0.0499 (12)	
H34	0.1973	0.5780	0.5817	0.060*	
C35	0.2586 (3)	0.6076 (2)	0.6958 (3)	0.0417 (11)	
H35	0.1992	0.6124	0.7106	0.050*	
C36	0.22181 (19)	0.83159 (16)	0.70249 (15)	0.0148 (5)	
C37	0.15190 (18)	0.75914 (17)	0.66475 (16)	0.0166 (5)	
H37	0.1218	0.7226	0.6872	0.020*	
C38	0.13525 (19)	0.75110 (19)	0.58924 (16)	0.0194 (6)	
H38	0.0946	0.7070	0.5524	0.023*	
C39	0.1891 (2)	0.81971 (18)	0.57744 (16)	0.0195 (5)	
H39	0.1915	0.8296	0.5314	0.023*	
C40	0.2386 (2)	0.87093 (17)	0.64555 (15)	0.0162 (5)	
H40	0.2770	0.9230	0.6529	0.019*	
C41	0.30012 (19)	0.83558 (17)	0.77373 (16)	0.0165 (5)	
C42	0.3601 (2)	0.92080 (19)	0.80907 (17)	0.0222 (6)	
H42	0.3795	0.9464	0.7715	0.027*	
C43	0.4583 (2)	0.9108 (2)	0.87292 (18)	0.0302 (7)	
H43A	0.4959	0.9665	0.8950	0.036*	
H43B	0.5048	0.8737	0.8547	0.036*	
C44	0.4288 (2)	0.8724 (2)	0.93092 (18)	0.0316 (7)	
H44	0.4932	0.8655	0.9723	0.038*	
C45	0.3719 (2)	0.7856 (2)	0.89559 (18)	0.0280 (7)	
H45A	0.4182	0.7482	0.8775	0.034*	
H45B	0.3532	0.7596	0.9326	0.034*	
C46	0.2733 (2)	0.79519 (19)	0.83160 (16)	0.0195 (6)	
H46	0.2361	0.7387	0.8090	0.023*	
C47	0.2898 (2)	0.97955 (19)	0.83913 (18)	0.0265 (7)	
H47A	0.3271	1.0354	0.8615	0.032*	
H47B	0.2263	0.9877	0.7985	0.032*	
C48	0.3582 (3)	0.9296 (2)	0.96019 (19)	0.0323 (8)	
H48A	0.3393	0.9044	0.9976	0.039*	
H48B	0.3951	0.9853	0.9837	0.039*	
C49	0.2023 (2)	0.85360 (19)	0.86180 (17)	0.0226 (6)	
H49A	0.1377	0.8602	0.8214	0.027*	
H49B	0.1831	0.8281	0.8989	0.027*	
C50	0.2600 (2)	0.9406 (2)	0.89689 (18)	0.0266 (7)	
H50	0.2139	0.9784	0.9159	0.032*	
Zr3	0.17259 (2)	0.02164 (2)	0.35641 (2)	0.01183 (5)	
C51	0.2177 (3)	0.15654 (19)	0.3091 (2)	0.0295 (7)	
H51	0.2407	0.1475	0.2680	0.035*	
C52	0.2799 (2)	0.16506 (18)	0.3813 (2)	0.0263 (7)	
H52	0.3535	0.1650	0.3987	0.032*	
C53	0.2165 (2)	0.17380 (18)	0.42502 (18)	0.0233 (6)	
H53	0.2390	0.1769	0.4768	0.028*	
C54	0.1140 (2)	0.17722 (18)	0.3784 (2)	0.0243 (7)	
H54	0.0552	0.1871	0.3934	0.029*	
C55	0.1135 (2)	0.16350 (19)	0.3063 (2)	0.0286 (7)	
H55	0.0541	0.1596	0.2632	0.034*	
C56	−0.0062 (2)	−0.0617 (2)	0.3120 (2)	0.0342 (9)	
H56	−0.0303	−0.0868	0.2617	0.041*	
C57	−0.0243 (2)	0.02045 (19)	0.34153 (19)	0.0242 (7)	
H57	−0.0646	0.0602	0.3148	0.029*	
C58	0.0274 (2)	0.0321 (2)	0.41629 (19)	0.0266 (7)	
H58	0.0281	0.0816	0.4498	0.032*	
C59	0.0777 (3)	−0.0391 (2)	0.4347 (2)	0.0337 (8)	
H59	0.1209	−0.0462	0.4825	0.040*	
C60	0.0545 (2)	−0.09897 (19)	0.3716 (2)	0.0370 (10)	
H60	0.0758	−0.1551	0.3691	0.044*	
C61	0.2692 (2)	−0.06680 (17)	0.30162 (17)	0.0183 (6)	
C62	0.2524 (2)	−0.11713 (18)	0.35200 (17)	0.0202 (6)	
H62	0.2097	−0.1680	0.3385	0.024*	
C63	0.3090 (2)	−0.0795 (2)	0.42394 (19)	0.0248 (6)	
H63	0.3086	−0.0989	0.4673	0.030*	
C64	0.3669 (2)	−0.0073 (2)	0.42099 (19)	0.0249 (6)	
H64	0.4116	0.0303	0.4619	0.030*	
C65	0.3463 (2)	−0.00141 (19)	0.34688 (18)	0.0224 (6)	
H65	0.3783	0.0394	0.3292	0.027*	
C66	0.1889 (2)	−0.05370 (18)	0.23524 (17)	0.0196 (6)	
C67	0.2210 (3)	−0.0062 (2)	0.18306 (19)	0.0269 (7)	
H67	0.2693	0.0435	0.2120	0.032*	
C68	0.1258 (3)	0.0237 (2)	0.1271 (2)	0.0364 (8)	
H68A	0.0898	0.0635	0.1530	0.044*	
H68B	0.1482	0.0538	0.0938	0.044*	
C69	0.0517 (3)	−0.0514 (3)	0.0829 (2)	0.0415 (9)	
H69	−0.0107	−0.0314	0.0470	0.050*	
C70	0.0179 (3)	−0.0972 (2)	0.1348 (2)	0.0365 (8)	
H70A	−0.0307	−0.1460	0.1063	0.044*	
H70B	−0.0188	−0.0587	0.1610	0.044*	
C71	0.1129 (2)	−0.1280 (2)	0.19077 (18)	0.0257 (6)	
H71	0.0901	−0.1583	0.2244	0.031*	
C72	0.2770 (3)	−0.0669 (2)	0.1411 (2)	0.0370 (9)	
H72A	0.3000	−0.0370	0.1079	0.044*	
H72B	0.3391	−0.0866	0.1764	0.044*	
C73	0.1075 (4)	−0.1122 (3)	0.0413 (2)	0.0483 (11)	
H73A	0.0593	−0.1610	0.0119	0.058*	
H73B	0.1296	−0.0828	0.0074	0.058*	
C74	0.1684 (3)	−0.1886 (2)	0.1486 (2)	0.0331 (8)	
H74A	0.2301	−0.2088	0.1838	0.040*	
H74B	0.1208	−0.2381	0.1205	0.040*	
C75	0.2020 (3)	−0.1429 (2)	0.0962 (2)	0.0401 (9)	
H75	0.2381	−0.1823	0.0694	0.048*	
Zr4	0.16561 (2)	0.52009 (2)	0.35633 (2)	0.01146 (5)	
C76	0.2956 (2)	0.41870 (19)	0.3221 (2)	0.0279 (7)	
H76	0.2749	0.3925	0.2722	0.034*	
C77	0.2735 (2)	0.38672 (19)	0.3785 (2)	0.0290 (7)	
H77	0.2381	0.3340	0.3739	0.035*	
C78	0.3130 (2)	0.4463 (2)	0.44286 (19)	0.0263 (7)	
H78	0.3061	0.4424	0.4890	0.032*	
C79	0.3649 (2)	0.51291 (19)	0.42664 (18)	0.0223 (6)	
H79	0.4011	0.5610	0.4606	0.027*	
C80	0.3538 (2)	0.49636 (19)	0.35210 (19)	0.0241 (6)	
H80	0.3807	0.5311	0.3265	0.029*	
C81	0.2010 (3)	0.65239 (19)	0.30234 (18)	0.0259 (7)	
H81	0.2182	0.6415	0.2588	0.031*	
C82	0.2712 (2)	0.66405 (17)	0.37391 (18)	0.0216 (6)	
H82	0.3447	0.6655	0.3877	0.026*	
C83	0.2138 (2)	0.67337 (17)	0.42211 (17)	0.0201 (6)	
H83	0.2415	0.6782	0.4740	0.024*	
C84	0.1083 (2)	0.67435 (18)	0.37968 (19)	0.0237 (6)	
H84	0.0525	0.6834	0.3981	0.028*	
C85	0.0999 (2)	0.65966 (19)	0.30595 (19)	0.0267 (7)	
H85	0.0373	0.6553	0.2653	0.032*	
C86	0.00649 (18)	0.44439 (17)	0.29543 (16)	0.0164 (5)	
C87	−0.02264 (19)	0.51655 (18)	0.33577 (16)	0.0178 (5)	
H87	−0.0615	0.5607	0.3150	0.021*	
C88	0.0155 (2)	0.5111 (2)	0.41062 (17)	0.0226 (6)	
H88	0.0103	0.5523	0.4493	0.027*	
C89	0.0627 (2)	0.4343 (2)	0.41867 (17)	0.0223 (6)	
H89	0.0945	0.4144	0.4637	0.027*	
C90	0.0549 (2)	0.39190 (17)	0.34873 (17)	0.0195 (6)	
H90	0.0779	0.3373	0.3384	0.023*	
C91	0.0358 (2)	0.44995 (18)	0.23175 (16)	0.0194 (6)	
C92	0.0661 (2)	0.36983 (19)	0.19246 (18)	0.0241 (6)	
H92	0.1139	0.3387	0.2292	0.029*	
C93	0.1192 (3)	0.3925 (2)	0.1394 (2)	0.0361 (9)	
H93A	0.1837	0.4282	0.1672	0.043*	
H93B	0.1383	0.3404	0.1142	0.043*	
C94	0.0470 (3)	0.4397 (3)	0.0825 (2)	0.0421 (10)	
H94	0.0828	0.4544	0.0485	0.051*	
C95	0.0195 (3)	0.5201 (2)	0.12220 (19)	0.0365 (8)	
H95A	−0.0272	0.5515	0.0859	0.044*	
H95B	0.0834	0.5565	0.1499	0.044*	
C96	−0.0342 (2)	0.4987 (2)	0.17498 (18)	0.0259 (6)	
H96	−0.0525	0.5518	0.2005	0.031*	
C97	−0.0340 (2)	0.3136 (2)	0.1473 (2)	0.0322 (8)	
H97A	−0.0157	0.2611	0.1220	0.039*	
H97B	−0.0695	0.2979	0.1806	0.039*	
C98	−0.0529 (3)	0.3835 (3)	0.0383 (2)	0.0464 (10)	
H98A	−0.1002	0.4138	0.0014	0.056*	
H98B	−0.0352	0.3313	0.0122	0.056*	
C99	−0.1345 (3)	0.4425 (2)	0.1305 (2)	0.0351 (8)	
H99A	−0.1706	0.4285	0.1638	0.042*	
H99B	−0.1819	0.4735	0.0941	0.042*	
C100	−0.1066 (3)	0.3612 (2)	0.0908 (2)	0.0380 (9)	
H100	−0.1714	0.3250	0.0624	0.046*	
C101	0.2208 (8)	0.2642 (6)	−0.0633 (9)	0.050 (2)	0.5
C102	0.2427 (5)	0.1823 (5)	−0.0513 (6)	0.0411 (18)	0.5
H102	0.2244	0.1372	−0.0915	0.049*	0.5
C103	0.2883 (7)	0.1677 (6)	0.0150 (6)	0.057 (2)	0.5
H103	0.2962	0.1109	0.0229	0.068*	0.5
C104	0.3251 (10)	0.2308 (9)	0.0735 (9)	0.077 (4)	0.5
H104	0.3608	0.2203	0.1218	0.092*	0.5
C105	0.3052 (7)	0.3174 (6)	0.0563 (7)	0.062 (3)	0.5
H105	0.3317	0.3638	0.0950	0.074*	0.5
C106	0.2523 (10)	0.3332 (6)	−0.0101 (9)	0.058 (3)	0.5
H106	0.2376	0.3887	−0.0198	0.070*	0.5
C107	0.1697 (15)	0.2892 (17)	−0.1291 (12)	0.126 (7)	0.5
H10F	0.1629	0.3503	−0.1213	0.189*	0.5
H10G	0.1005	0.2600	−0.1506	0.189*	0.5
H10H	0.2092	0.2754	−0.1624	0.189*	0.5
C108	0.2677 (9)	0.2072 (9)	0.1250 (9)	0.107 (5)	0.5
H10A	0.2311	0.2368	0.1559	0.160*	0.5
H10B	0.3106	0.1656	0.1509	0.160*	0.5
H10C	0.2169	0.1784	0.0790	0.160*	0.5
C109	0.3356 (8)	0.2694 (8)	0.1086 (7)	0.065 (3)	0.5
H10D	0.3155	0.3273	0.1208	0.078*	0.5
H10E	0.4089	0.2656	0.1390	0.078*	0.5
C110	0.3242 (14)	0.2513 (13)	0.0296 (10)	0.115 (6)	0.5
H11A	0.3894	0.2704	0.0234	0.138*	0.5
H11B	0.3132	0.1896	0.0126	0.138*	0.5
C111	0.2397 (13)	0.2919 (13)	−0.0149 (9)	0.092 (5)	0.5
H11C	0.2572	0.3530	−0.0095	0.111*	0.5
H11D	0.1763	0.2840	−0.0022	0.111*	0.5
C112	0.2243 (10)	0.2438 (11)	−0.0980 (8)	0.083 (4)	0.5
H11E	0.2881	0.2525	−0.1100	0.100*	0.5
H11F	0.2098	0.1825	−0.1019	0.100*	0.5
C113	0.1355 (10)	0.2802 (9)	−0.1492 (6)	0.046 (2)	0.5
H11G	0.0781	0.2844	−0.1297	0.069*	0.5
H11H	0.1127	0.2437	−0.1972	0.069*	0.5
H11I	0.1572	0.3365	−0.1544	0.069*	0.5

### Atomic displacement parameters (Å^2^)

**Table:**

	*U*^11^	*U*^22^	*U*^33^	*U*^12^	*U*^13^	*U*^23^
Zr1	0.01101 (10)	0.00916 (11)	0.01929 (14)	0.00180 (8)	0.00651 (10)	0.00375 (10)
C1	0.0270 (14)	0.0175 (15)	0.037 (2)	0.0013 (11)	0.0042 (13)	0.0142 (14)
C2	0.0225 (13)	0.0138 (14)	0.061 (2)	0.0082 (10)	0.0222 (15)	0.0144 (15)
C3	0.0317 (15)	0.0100 (12)	0.0349 (18)	0.0012 (10)	0.0178 (13)	0.0030 (11)
C4	0.0185 (12)	0.0142 (13)	0.043 (2)	−0.0006 (10)	0.0150 (13)	0.0071 (13)
C5	0.0326 (15)	0.0188 (15)	0.0362 (19)	0.0034 (11)	0.0209 (14)	0.0128 (13)
C6	0.0154 (11)	0.0207 (14)	0.0286 (17)	0.0068 (10)	0.0069 (11)	−0.0012 (12)
C7	0.0126 (11)	0.0178 (14)	0.0348 (18)	0.0027 (9)	0.0092 (11)	0.0077 (12)
C8	0.0135 (11)	0.0214 (15)	0.0298 (17)	0.0037 (10)	0.0021 (11)	0.0016 (13)
C9	0.0187 (12)	0.0278 (16)	0.0337 (18)	0.0105 (11)	0.0080 (12)	0.0145 (14)
C10	0.0153 (11)	0.0129 (13)	0.048 (2)	0.0046 (9)	0.0106 (13)	0.0084 (13)
C11	0.0108 (10)	0.0124 (12)	0.0214 (14)	0.0000 (8)	0.0056 (10)	0.0024 (10)
C12	0.0142 (11)	0.0112 (12)	0.0247 (15)	−0.0008 (8)	0.0082 (10)	0.0039 (10)
C13	0.0156 (11)	0.0185 (13)	0.0178 (14)	−0.0023 (9)	0.0046 (10)	0.0033 (11)
C14	0.0150 (11)	0.0169 (13)	0.0223 (15)	−0.0014 (9)	0.0097 (10)	0.0010 (11)
C15	0.0107 (10)	0.0146 (12)	0.0239 (15)	0.0010 (8)	0.0081 (10)	0.0036 (11)
C16	0.0154 (11)	0.0191 (13)	0.0181 (14)	0.0041 (9)	0.0063 (10)	0.0016 (11)
C17	0.0198 (12)	0.0227 (15)	0.0173 (14)	0.0052 (10)	0.0050 (11)	0.0032 (11)
C18	0.0286 (15)	0.0373 (19)	0.0205 (16)	0.0072 (13)	0.0085 (13)	0.0095 (14)
C19	0.0316 (16)	0.048 (2)	0.0198 (17)	0.0105 (15)	0.0118 (13)	0.0015 (15)
C20	0.0266 (15)	0.0396 (19)	0.0239 (17)	0.0113 (13)	0.0100 (13)	−0.0023 (14)
C21	0.0227 (13)	0.0214 (15)	0.0216 (16)	0.0072 (11)	0.0064 (11)	0.0001 (12)
C22	0.0192 (12)	0.0288 (17)	0.0217 (16)	0.0049 (11)	0.0045 (11)	−0.0005 (13)
C23	0.0331 (16)	0.039 (2)	0.0194 (17)	0.0084 (14)	0.0029 (13)	−0.0061 (15)
C24	0.0315 (15)	0.0203 (15)	0.0277 (18)	0.0040 (12)	0.0071 (13)	−0.0034 (13)
C25	0.0254 (14)	0.0284 (18)	0.0258 (18)	0.0015 (12)	0.0045 (13)	−0.0049 (14)
Zr2	0.01016 (10)	0.00995 (11)	0.01683 (13)	0.00053 (8)	0.00541 (9)	0.00342 (10)
C26	0.0283 (15)	0.051 (2)	0.0274 (19)	−0.0275 (15)	0.0169 (14)	−0.0126 (16)
C27	0.0225 (13)	0.0207 (15)	0.048 (2)	0.0009 (11)	0.0193 (14)	0.0150 (14)
C28	0.0214 (13)	0.0385 (19)	0.0226 (16)	−0.0117 (12)	0.0111 (12)	−0.0015 (14)
C29	0.0312 (16)	0.0276 (17)	0.075 (3)	0.0058 (13)	0.040 (2)	0.0155 (18)
C30	0.0115 (12)	0.073 (3)	0.050 (2)	−0.0005 (15)	0.0089 (14)	0.038 (2)
C31	0.069 (3)	0.0175 (15)	0.033 (2)	0.0068 (15)	0.0220 (19)	0.0119 (14)
C32	0.0271 (16)	0.0268 (18)	0.079 (3)	0.0139 (13)	0.0219 (19)	0.029 (2)
C33	0.101 (4)	0.0120 (15)	0.056 (3)	0.0115 (18)	0.059 (3)	0.0083 (16)
C34	0.054 (2)	0.0108 (16)	0.061 (3)	−0.0111 (15)	−0.013 (2)	0.0071 (17)
C35	0.0412 (19)	0.0165 (15)	0.091 (4)	0.0084 (13)	0.048 (2)	0.0222 (19)
C36	0.0141 (10)	0.0109 (12)	0.0195 (14)	0.0013 (8)	0.0066 (10)	0.0006 (10)
C37	0.0109 (10)	0.0165 (13)	0.0222 (15)	0.0016 (9)	0.0062 (10)	0.0014 (11)
C38	0.0138 (11)	0.0226 (14)	0.0176 (14)	0.0033 (10)	0.0019 (10)	−0.0017 (11)
C39	0.0204 (12)	0.0201 (14)	0.0186 (14)	0.0098 (10)	0.0060 (11)	0.0051 (11)
C40	0.0190 (11)	0.0128 (12)	0.0183 (14)	0.0054 (9)	0.0075 (10)	0.0043 (10)
C41	0.0157 (11)	0.0155 (13)	0.0179 (14)	−0.0033 (9)	0.0060 (10)	0.0012 (10)
C42	0.0256 (13)	0.0209 (14)	0.0188 (15)	−0.0105 (11)	0.0085 (11)	−0.0022 (12)
C43	0.0242 (14)	0.042 (2)	0.0197 (16)	−0.0152 (13)	0.0050 (12)	−0.0020 (14)
C44	0.0265 (15)	0.045 (2)	0.0178 (16)	−0.0069 (14)	0.0018 (12)	0.0026 (15)
C45	0.0320 (15)	0.0330 (18)	0.0203 (16)	0.0007 (13)	0.0084 (13)	0.0097 (14)
C46	0.0220 (12)	0.0194 (14)	0.0178 (14)	−0.0039 (10)	0.0078 (11)	0.0025 (11)
C47	0.0362 (16)	0.0173 (14)	0.0244 (17)	−0.0071 (12)	0.0110 (13)	−0.0031 (12)
C48	0.0362 (17)	0.039 (2)	0.0190 (17)	−0.0152 (14)	0.0099 (14)	−0.0044 (15)
C49	0.0242 (13)	0.0226 (15)	0.0220 (16)	−0.0065 (11)	0.0120 (12)	−0.0029 (12)
C50	0.0316 (15)	0.0217 (16)	0.0257 (17)	−0.0086 (12)	0.0141 (13)	−0.0077 (13)
Zr3	0.01074 (10)	0.00897 (11)	0.01757 (13)	0.00172 (8)	0.00685 (9)	0.00275 (9)
C51	0.0496 (19)	0.0124 (14)	0.036 (2)	−0.0010 (12)	0.0284 (17)	0.0020 (13)
C52	0.0173 (12)	0.0122 (13)	0.050 (2)	−0.0018 (10)	0.0132 (13)	0.0039 (14)
C53	0.0305 (14)	0.0122 (13)	0.0247 (17)	−0.0042 (11)	0.0081 (13)	−0.0013 (12)
C54	0.0213 (13)	0.0104 (13)	0.047 (2)	0.0022 (10)	0.0197 (14)	0.0032 (13)
C55	0.0314 (15)	0.0122 (14)	0.0326 (19)	−0.0019 (11)	−0.0032 (13)	0.0073 (13)
C56	0.0212 (14)	0.042 (2)	0.039 (2)	−0.0178 (13)	0.0184 (14)	−0.0141 (16)
C57	0.0123 (11)	0.0239 (15)	0.042 (2)	0.0035 (10)	0.0122 (12)	0.0133 (14)
C58	0.0259 (14)	0.0272 (16)	0.0342 (19)	−0.0019 (12)	0.0219 (14)	0.0007 (14)
C59	0.0302 (16)	0.044 (2)	0.040 (2)	0.0052 (14)	0.0217 (15)	0.0230 (17)
C60	0.0292 (15)	0.0121 (14)	0.085 (3)	0.0031 (11)	0.0368 (19)	0.0135 (17)
C61	0.0165 (11)	0.0135 (12)	0.0295 (16)	0.0053 (9)	0.0148 (11)	0.0010 (11)
C62	0.0189 (12)	0.0144 (13)	0.0314 (17)	0.0070 (10)	0.0122 (12)	0.0072 (12)
C63	0.0219 (13)	0.0240 (15)	0.0307 (18)	0.0106 (11)	0.0085 (12)	0.0112 (13)
C64	0.0150 (12)	0.0263 (16)	0.0308 (18)	0.0064 (10)	0.0038 (12)	0.0058 (13)
C65	0.0132 (11)	0.0202 (14)	0.0377 (19)	0.0033 (10)	0.0133 (12)	0.0058 (13)
C66	0.0243 (13)	0.0161 (13)	0.0221 (15)	0.0063 (10)	0.0135 (12)	0.0014 (11)
C67	0.0404 (17)	0.0208 (15)	0.0267 (17)	0.0073 (12)	0.0206 (14)	0.0041 (13)
C68	0.057 (2)	0.035 (2)	0.0243 (18)	0.0160 (16)	0.0213 (17)	0.0064 (15)
C69	0.056 (2)	0.045 (2)	0.0195 (18)	0.0146 (18)	0.0081 (17)	0.0051 (16)
C70	0.0372 (18)	0.040 (2)	0.0255 (19)	0.0024 (15)	0.0056 (15)	−0.0041 (16)
C71	0.0312 (15)	0.0217 (15)	0.0236 (16)	−0.0004 (12)	0.0111 (13)	−0.0027 (12)
C72	0.057 (2)	0.0297 (19)	0.040 (2)	0.0103 (16)	0.0371 (19)	0.0060 (16)
C73	0.080 (3)	0.041 (2)	0.028 (2)	0.008 (2)	0.026 (2)	−0.0011 (18)
C74	0.048 (2)	0.0225 (17)	0.0300 (19)	0.0049 (14)	0.0192 (16)	−0.0050 (14)
C75	0.065 (2)	0.032 (2)	0.032 (2)	0.0141 (17)	0.0308 (19)	−0.0003 (16)
Zr4	0.01103 (10)	0.00883 (11)	0.01512 (13)	0.00078 (8)	0.00556 (9)	0.00119 (9)
C76	0.0174 (12)	0.0202 (15)	0.042 (2)	0.0051 (10)	0.0097 (13)	−0.0080 (14)
C77	0.0152 (12)	0.0145 (14)	0.056 (2)	0.0041 (10)	0.0095 (14)	0.0077 (14)
C78	0.0182 (12)	0.0272 (16)	0.0357 (19)	0.0060 (11)	0.0073 (12)	0.0160 (14)
C79	0.0137 (11)	0.0221 (15)	0.0281 (17)	0.0006 (10)	0.0032 (11)	0.0033 (12)
C80	0.0142 (11)	0.0229 (15)	0.0384 (19)	0.0026 (10)	0.0136 (12)	0.0037 (13)
C81	0.0452 (18)	0.0130 (14)	0.0231 (16)	−0.0045 (12)	0.0168 (14)	0.0020 (12)
C82	0.0210 (12)	0.0116 (13)	0.0348 (18)	−0.0014 (10)	0.0135 (12)	0.0022 (12)
C83	0.0232 (13)	0.0134 (13)	0.0212 (15)	−0.0016 (10)	0.0068 (11)	−0.0026 (11)
C84	0.0202 (12)	0.0118 (13)	0.0387 (19)	0.0015 (10)	0.0114 (13)	−0.0003 (12)
C85	0.0286 (14)	0.0130 (14)	0.0289 (18)	−0.0022 (11)	−0.0039 (13)	0.0067 (13)
C86	0.0102 (10)	0.0137 (12)	0.0246 (15)	−0.0031 (8)	0.0051 (10)	0.0029 (11)
C87	0.0123 (10)	0.0184 (13)	0.0260 (16)	0.0025 (9)	0.0090 (10)	0.0072 (11)
C88	0.0195 (12)	0.0271 (16)	0.0270 (16)	0.0031 (11)	0.0149 (12)	0.0054 (13)
C89	0.0195 (12)	0.0282 (16)	0.0251 (16)	0.0027 (11)	0.0106 (11)	0.0146 (13)
C90	0.0167 (11)	0.0134 (12)	0.0319 (17)	0.0002 (9)	0.0108 (11)	0.0080 (11)
C91	0.0189 (12)	0.0178 (14)	0.0201 (15)	−0.0058 (10)	0.0066 (11)	−0.0009 (11)
C92	0.0249 (14)	0.0213 (15)	0.0239 (16)	−0.0087 (11)	0.0102 (12)	−0.0069 (12)
C93	0.0405 (18)	0.040 (2)	0.0284 (19)	−0.0181 (15)	0.0214 (16)	−0.0151 (16)
C94	0.059 (2)	0.046 (2)	0.0190 (18)	−0.0270 (18)	0.0164 (17)	−0.0082 (16)
C95	0.047 (2)	0.037 (2)	0.0199 (17)	−0.0193 (16)	0.0041 (15)	0.0050 (15)
C96	0.0285 (14)	0.0235 (16)	0.0192 (16)	−0.0075 (12)	−0.0004 (12)	0.0035 (12)
C97	0.0335 (16)	0.0238 (17)	0.033 (2)	−0.0135 (13)	0.0094 (15)	−0.0082 (15)
C98	0.060 (2)	0.047 (2)	0.0222 (19)	−0.0241 (19)	0.0063 (18)	−0.0071 (17)
C99	0.0296 (16)	0.036 (2)	0.0288 (19)	−0.0106 (14)	−0.0046 (14)	0.0076 (15)
C100	0.0388 (18)	0.036 (2)	0.0271 (19)	−0.0216 (15)	0.0002 (15)	−0.0054 (16)
C101	0.041 (4)	0.032 (4)	0.093 (7)	0.004 (3)	0.044 (5)	0.013 (5)
C102	0.028 (3)	0.020 (3)	0.074 (5)	0.006 (2)	0.019 (3)	0.000 (3)
C103	0.050 (5)	0.036 (5)	0.071 (6)	0.004 (4)	0.010 (4)	−0.008 (4)
C104	0.066 (7)	0.067 (7)	0.067 (7)	0.026 (5)	−0.006 (6)	−0.015 (5)
C105	0.048 (5)	0.040 (5)	0.095 (7)	−0.002 (4)	0.034 (5)	−0.017 (5)
C106	0.055 (5)	0.020 (4)	0.107 (8)	−0.008 (4)	0.042 (5)	0.001 (4)
C107	0.090 (13)	0.167 (17)	0.145 (12)	−0.007 (11)	0.045 (9)	0.083 (11)
C108	0.072 (8)	0.101 (11)	0.146 (13)	−0.006 (7)	0.015 (8)	0.067 (10)
C109	0.038 (5)	0.063 (7)	0.088 (8)	−0.005 (5)	0.009 (5)	0.023 (6)
C110	0.114 (11)	0.130 (15)	0.104 (9)	0.010 (9)	0.028 (8)	0.049 (9)
C111	0.079 (9)	0.152 (16)	0.058 (7)	0.031 (10)	0.035 (6)	0.025 (9)
C112	0.052 (6)	0.133 (13)	0.083 (8)	0.016 (7)	0.045 (6)	0.021 (8)
C113	0.057 (6)	0.048 (5)	0.034 (5)	−0.009 (4)	0.019 (4)	0.003 (4)

### Geometric parameters (Å, º)

**Table:**

Zr1—C11	2.334 (2)	C51—C55	1.413 (4)
Zr1—C15	2.482 (2)	C51—H51	0.9500
Zr1—C12	2.487 (2)	C52—C53	1.401 (4)
Zr1—C3	2.578 (3)	C52—H52	0.9500
Zr1—C1	2.601 (3)	C53—C54	1.403 (4)
Zr1—C9	2.604 (3)	C53—H53	0.9500
Zr1—C16	2.605 (3)	C54—C55	1.395 (5)
Zr1—C6	2.613 (3)	C54—H54	0.9500
Zr1—C5	2.614 (3)	C55—H55	0.9500
Zr1—C10	2.615 (3)	C56—C60	1.410 (6)
Zr1—C8	2.619 (3)	C56—C57	1.418 (5)
Zr1—C2	2.620 (3)	C56—H56	0.9500
Zr1—C14	2.620 (2)	C57—C58	1.380 (5)
Zr1—C13	2.623 (3)	C57—H57	0.9500
Zr1—C4	2.631 (3)	C58—C59	1.373 (5)
Zr1—C7	2.653 (2)	C58—H58	0.9500
C1—C2	1.397 (5)	C59—C60	1.389 (5)
C1—C5	1.415 (4)	C59—H59	0.9500
C1—H1	0.9500	C60—H60	0.9500
C2—C3	1.409 (5)	C61—C62	1.439 (4)
C2—H2	0.9500	C61—C65	1.441 (4)
C3—C4	1.412 (4)	C61—C66	1.442 (4)
C3—H3	0.9500	C62—C63	1.400 (5)
C4—C5	1.383 (5)	C62—H62	0.9500
C4—H4	0.9500	C63—C64	1.417 (4)
C5—H5	0.9500	C63—H63	0.9500
C6—C10	1.411 (4)	C64—C65	1.402 (5)
C6—C7	1.412 (4)	C64—H64	0.9500
C6—H6	0.9500	C65—H65	0.9500
C7—C8	1.399 (4)	C66—C71	1.524 (4)
C7—H7	0.9500	C66—C67	1.532 (4)
C8—C9	1.412 (4)	C67—C68	1.533 (5)
C8—H8	0.9500	C67—C72	1.551 (4)
C9—C10	1.400 (5)	C67—H67	1.0000
C9—H9	0.9500	C68—C69	1.520 (5)
C10—H10	0.9500	C68—H68A	0.9900
C11—C15	1.437 (4)	C68—H68B	0.9900
C11—C12	1.437 (4)	C69—C70	1.524 (5)
C11—C16	1.448 (4)	C69—C73	1.546 (5)
C12—C13	1.406 (4)	C69—H69	1.0000
C12—H12	0.9500	C70—C71	1.537 (5)
C13—C14	1.408 (4)	C70—H70A	0.9900
C13—H13	0.9500	C70—H70B	0.9900
C14—C15	1.406 (4)	C71—C74	1.547 (4)
C14—H14	0.9500	C71—H71	1.0000
C15—H15	0.9500	C72—C75	1.538 (5)
C16—C17	1.523 (4)	C72—H72A	0.9900
C16—C21	1.535 (4)	C72—H72B	0.9900
C17—C18	1.536 (4)	C73—C75	1.522 (6)
C17—C22	1.547 (4)	C73—H73A	0.9900
C17—H17	1.0000	C73—H73B	0.9900
C18—C19	1.531 (5)	C74—C75	1.528 (5)
C18—H18A	0.9900	C74—H74A	0.9900
C18—H18B	0.9900	C74—H74B	0.9900
C19—C23	1.527 (5)	C75—H75	1.0000
C19—C20	1.538 (5)	Zr4—C86	2.329 (2)
C19—H19	1.0000	Zr4—C87	2.468 (2)
C20—C21	1.538 (4)	Zr4—C90	2.502 (3)
C20—H20A	0.9900	Zr4—C83	2.571 (3)
C20—H20B	0.9900	Zr4—C91	2.574 (3)
C21—C24	1.547 (4)	Zr4—C78	2.594 (3)
C21—H21	1.0000	Zr4—C76	2.605 (3)
C22—C25	1.534 (4)	Zr4—C88	2.619 (2)
C22—H22A	0.9900	Zr4—C81	2.622 (3)
C22—H22B	0.9900	Zr4—C77	2.623 (3)
C23—C25	1.534 (5)	Zr4—C79	2.625 (3)
C23—H23A	0.9900	Zr4—C84	2.628 (3)
C23—H23B	0.9900	Zr4—C82	2.631 (3)
C24—C25	1.523 (5)	Zr4—C80	2.632 (2)
C24—H24A	0.9900	Zr4—C85	2.633 (3)
C24—H24B	0.9900	Zr4—C89	2.643 (3)
C25—H25	1.0000	C76—C77	1.402 (5)
Zr2—C36	2.337 (2)	C76—C80	1.409 (4)
Zr2—C40	2.470 (3)	C76—H76	0.9500
Zr2—C37	2.499 (2)	C77—C78	1.404 (5)
Zr2—C34	2.573 (3)	C77—H77	0.9500
Zr2—C33	2.600 (3)	C78—C79	1.410 (4)
Zr2—C28	2.602 (3)	C78—H78	0.9500
Zr2—C26	2.603 (3)	C79—C80	1.400 (4)
Zr2—C39	2.606 (3)	C79—H79	0.9500
Zr2—C41	2.609 (3)	C80—H80	0.9500
Zr2—C35	2.612 (3)	C81—C82	1.394 (5)
Zr2—C31	2.613 (3)	C81—C85	1.413 (4)
Zr2—C30	2.614 (3)	C81—H81	0.9500
Zr2—C38	2.628 (3)	C82—C83	1.407 (4)
Zr2—C27	2.630 (3)	C82—H82	0.9500
Zr2—C29	2.633 (3)	C83—C84	1.408 (4)
Zr2—C32	2.641 (3)	C83—H83	0.9500
C26—C27	1.385 (5)	C84—C85	1.393 (5)
C26—C30	1.394 (6)	C84—H84	0.9500
C26—H26	0.9500	C85—H85	0.9500
C27—C28	1.402 (5)	C86—C90	1.434 (4)
C27—H27	0.9500	C86—C87	1.439 (4)
C28—C29	1.392 (5)	C86—C91	1.446 (4)
C28—H28	0.9500	C87—C88	1.400 (4)
C29—C30	1.371 (6)	C87—H87	0.9500
C29—H29	0.9500	C88—C89	1.405 (4)
C30—H30	0.9500	C88—H88	0.9500
C31—C35	1.377 (6)	C89—C90	1.404 (4)
C31—C32	1.379 (5)	C89—H89	0.9500
C31—H31	0.9500	C90—H90	0.9500
C32—C33	1.369 (6)	C91—C96	1.529 (4)
C32—H32	0.9500	C91—C92	1.531 (4)
C33—C34	1.393 (6)	C92—C93	1.534 (4)
C33—H33	0.9500	C92—C97	1.549 (4)
C34—C35	1.376 (6)	C92—H92	1.0000
C34—H34	0.9500	C93—C94	1.526 (6)
C35—H35	0.9500	C93—H93A	0.9900
C36—C37	1.437 (4)	C93—H93B	0.9900
C36—C41	1.441 (4)	C94—C95	1.521 (5)
C36—C40	1.442 (4)	C94—C98	1.545 (5)
C37—C38	1.405 (4)	C94—H94	1.0000
C37—H37	0.9500	C95—C96	1.527 (4)
C38—C39	1.410 (4)	C95—H95A	0.9900
C38—H38	0.9500	C95—H95B	0.9900
C39—C40	1.405 (4)	C96—C99	1.548 (4)
C39—H39	0.9500	C96—H96	1.0000
C40—H40	0.9500	C97—C100	1.529 (5)
C41—C46	1.528 (4)	C97—H97A	0.9900
C41—C42	1.531 (4)	C97—H97B	0.9900
C42—C43	1.530 (5)	C98—C100	1.527 (5)
C42—C47	1.546 (4)	C98—H98A	0.9900
C42—H42	1.0000	C98—H98B	0.9900
C43—C44	1.532 (5)	C99—C100	1.535 (5)
C43—H43A	0.9900	C99—H99A	0.9900
C43—H43B	0.9900	C99—H99B	0.9900
C44—C48	1.528 (5)	C100—H100	1.0000
C44—C45	1.535 (5)	C101—C106	1.357 (18)
C44—H44	1.0000	C101—C107	1.37 (2)
C45—C46	1.533 (4)	C101—C102	1.390 (12)
C45—H45A	0.9900	C102—C103	1.298 (14)
C45—H45B	0.9900	C102—H102	0.9500
C46—C49	1.551 (4)	C103—C104	1.360 (15)
C46—H46	1.0000	C103—H103	0.9500
C47—C50	1.535 (4)	C104—C105	1.488 (19)
C47—H47A	0.9900	C104—H104	0.9500
C47—H47B	0.9900	C105—C106	1.33 (2)
C48—C50	1.528 (5)	C105—H105	0.9500
C48—H48A	0.9900	C106—H106	0.9500
C48—H48B	0.9900	C107—H10F	0.9800
C49—C50	1.541 (4)	C107—H10G	0.9800
C49—H49A	0.9900	C107—H10H	0.9800
C49—H49B	0.9900	C108—C109	1.498 (15)
C50—H50	1.0000	C108—H10A	0.9800
Zr3—C61	2.332 (2)	C108—H10B	0.9800
Zr3—C65	2.471 (2)	C108—H10C	0.9800
Zr3—C62	2.495 (3)	C109—C110	1.49 (2)
Zr3—C53	2.567 (3)	C109—H10D	0.9900
Zr3—C66	2.577 (3)	C109—H10E	0.9900
Zr3—C56	2.588 (3)	C110—C111	1.43 (2)
Zr3—C59	2.597 (3)	C110—H11A	0.9900
Zr3—C57	2.610 (2)	C110—H11B	0.9900
Zr3—C52	2.612 (3)	C111—C112	1.64 (2)
Zr3—C58	2.616 (3)	C111—H11C	0.9900
Zr3—C64	2.616 (3)	C111—H11D	0.9900
Zr3—C60	2.625 (3)	C112—C113	1.48 (2)
Zr3—C51	2.627 (3)	C112—H11E	0.9900
Zr3—C63	2.634 (3)	C112—H11F	0.9900
Zr3—C55	2.646 (3)	C113—H11G	0.9800
Zr3—C54	2.650 (3)	C113—H11H	0.9800
C51—C52	1.371 (5)	C113—H11I	0.9800
			
C11—Zr1—C15	34.54 (9)	C62—Zr3—C52	122.63 (9)
C11—Zr1—C12	34.50 (9)	C53—Zr3—C52	31.38 (9)
C15—Zr1—C12	54.73 (9)	C66—Zr3—C52	103.48 (10)
C11—Zr1—C3	126.33 (9)	C56—Zr3—C52	149.13 (11)
C15—Zr1—C3	92.23 (9)	C59—Zr3—C52	130.06 (12)
C12—Zr1—C3	135.13 (9)	C57—Zr3—C52	119.87 (9)
C11—Zr1—C1	98.03 (10)	C61—Zr3—C58	146.65 (10)
C15—Zr1—C1	79.87 (9)	C65—Zr3—C58	157.88 (11)
C12—Zr1—C1	131.46 (9)	C62—Zr3—C58	115.14 (10)
C3—Zr1—C1	52.17 (11)	C53—Zr3—C58	81.23 (10)
C11—Zr1—C9	110.83 (10)	C66—Zr3—C58	135.81 (10)
C15—Zr1—C9	127.83 (9)	C56—Zr3—C58	51.37 (10)
C12—Zr1—C9	77.99 (9)	C59—Zr3—C58	30.55 (10)
C3—Zr1—C9	110.24 (11)	C57—Zr3—C58	30.64 (10)
C1—Zr1—C9	150.55 (10)	C52—Zr3—C58	112.24 (10)
C11—Zr1—C16	33.52 (9)	C61—Zr3—C64	55.60 (10)
C15—Zr1—C16	59.31 (8)	C65—Zr3—C64	31.85 (10)
C12—Zr1—C16	60.17 (9)	C62—Zr3—C64	52.98 (10)
C3—Zr1—C16	132.33 (10)	C53—Zr3—C64	87.56 (10)
C1—Zr1—C16	83.66 (10)	C66—Zr3—C64	87.85 (10)
C9—Zr1—C16	117.42 (10)	C56—Zr3—C64	137.06 (12)
C11—Zr1—C6	106.60 (9)	C59—Zr3—C64	103.17 (11)
C15—Zr1—C6	141.11 (9)	C57—Zr3—C64	154.31 (10)
C12—Zr1—C6	94.71 (9)	C52—Zr3—C64	73.56 (10)
C3—Zr1—C6	125.77 (9)	C58—Zr3—C64	126.11 (11)
C1—Zr1—C6	115.03 (10)	C61—Zr3—C60	96.13 (10)
C9—Zr1—C6	51.85 (10)	C65—Zr3—C60	122.75 (10)
C16—Zr1—C6	85.69 (9)	C62—Zr3—C60	68.12 (9)
C11—Zr1—C5	125.43 (10)	C53—Zr3—C60	129.84 (10)
C15—Zr1—C5	111.34 (9)	C66—Zr3—C60	95.70 (11)
C12—Zr1—C5	159.73 (10)	C56—Zr3—C60	31.37 (12)
C3—Zr1—C5	51.83 (10)	C59—Zr3—C60	30.85 (12)
C1—Zr1—C5	31.50 (9)	C57—Zr3—C60	51.40 (10)
C9—Zr1—C5	119.68 (10)	C52—Zr3—C60	160.80 (12)
C16—Zr1—C5	100.67 (10)	C58—Zr3—C60	50.58 (10)
C6—Zr1—C5	89.99 (10)	C64—Zr3—C60	108.34 (11)
C11—Zr1—C10	92.94 (9)	C61—Zr3—C51	94.79 (10)
C15—Zr1—C10	123.24 (9)	C65—Zr3—C51	76.03 (10)
C12—Zr1—C10	69.00 (8)	C62—Zr3—C51	127.84 (9)
C3—Zr1—C10	137.93 (10)	C53—Zr3—C51	51.35 (10)
C1—Zr1—C10	146.14 (10)	C66—Zr3—C51	82.54 (10)
C9—Zr1—C10	31.11 (10)	C56—Zr3—C51	126.29 (13)
C16—Zr1—C10	88.26 (9)	C59—Zr3—C51	146.06 (11)
C6—Zr1—C10	31.31 (10)	C57—Zr3—C51	107.42 (10)
C5—Zr1—C10	120.21 (9)	C52—Zr3—C51	30.34 (11)
C11—Zr1—C8	141.75 (10)	C58—Zr3—C51	117.02 (10)
C15—Zr1—C8	153.01 (10)	C64—Zr3—C51	94.32 (11)
C12—Zr1—C8	109.33 (9)	C60—Zr3—C51	157.22 (12)
C3—Zr1—C8	86.62 (10)	C61—Zr3—C63	55.18 (10)
C1—Zr1—C8	119.21 (10)	C65—Zr3—C63	52.87 (10)
C9—Zr1—C8	31.38 (9)	C62—Zr3—C63	31.52 (10)
C16—Zr1—C8	136.36 (9)	C53—Zr3—C63	110.49 (10)
C6—Zr1—C8	51.55 (10)	C66—Zr3—C63	87.45 (10)
C5—Zr1—C8	88.99 (10)	C56—Zr3—C63	105.99 (12)
C10—Zr1—C8	51.39 (9)	C59—Zr3—C63	78.33 (10)
C11—Zr1—C2	98.90 (9)	C57—Zr3—C63	126.84 (10)
C15—Zr1—C2	68.29 (9)	C52—Zr3—C63	104.08 (10)
C12—Zr1—C2	121.92 (9)	C58—Zr3—C63	107.37 (10)
C3—Zr1—C2	31.45 (10)	C64—Zr3—C63	31.31 (9)
C1—Zr1—C2	31.05 (11)	C60—Zr3—C63	77.23 (10)
C9—Zr1—C2	141.18 (11)	C51—Zr3—C63	125.18 (10)
C16—Zr1—C2	101.20 (10)	C61—Zr3—C55	119.01 (11)
C6—Zr1—C2	141.32 (10)	C65—Zr3—C55	106.90 (10)
C5—Zr1—C2	51.35 (9)	C62—Zr3—C55	153.18 (10)
C10—Zr1—C2	168.14 (9)	C53—Zr3—C55	51.44 (11)
C8—Zr1—C2	117.29 (10)	C66—Zr3—C55	94.64 (10)
C11—Zr1—C14	55.37 (9)	C56—Zr3—C55	99.36 (12)
C15—Zr1—C14	31.84 (9)	C59—Zr3—C55	119.17 (11)
C12—Zr1—C14	52.94 (9)	C57—Zr3—C55	76.46 (10)
C3—Zr1—C14	82.72 (9)	C52—Zr3—C55	50.86 (10)
C1—Zr1—C14	97.79 (10)	C58—Zr3—C55	88.63 (11)
C9—Zr1—C14	103.26 (9)	C64—Zr3—C55	123.40 (10)
C16—Zr1—C14	87.24 (8)	C60—Zr3—C55	127.48 (10)
C6—Zr1—C14	145.34 (9)	C51—Zr3—C55	31.09 (10)
C5—Zr1—C14	124.67 (9)	C63—Zr3—C55	154.65 (10)
C10—Zr1—C14	114.65 (9)	C61—Zr3—C54	145.55 (9)
C8—Zr1—C14	121.68 (10)	C65—Zr3—C54	119.91 (9)
C2—Zr1—C14	73.34 (9)	C62—Zr3—C54	171.19 (10)
C11—Zr1—C13	55.30 (9)	C53—Zr3—C54	31.14 (10)
C15—Zr1—C13	52.91 (9)	C66—Zr3—C54	125.18 (10)
C12—Zr1—C13	31.79 (9)	C56—Zr3—C54	99.94 (10)
C3—Zr1—C13	105.38 (9)	C59—Zr3—C54	95.31 (10)
C1—Zr1—C13	128.67 (9)	C57—Zr3—C54	69.09 (9)
C9—Zr1—C13	75.52 (9)	C52—Zr3—C54	50.89 (8)
C16—Zr1—C13	87.61 (9)	C58—Zr3—C54	67.01 (9)
C6—Zr1—C13	114.57 (9)	C64—Zr3—C54	118.49 (10)
C5—Zr1—C13	154.74 (9)	C60—Zr3—C54	116.25 (9)
C10—Zr1—C13	83.54 (9)	C51—Zr3—C54	50.77 (9)
C8—Zr1—C13	101.47 (9)	C63—Zr3—C54	140.09 (11)
C2—Zr1—C13	103.79 (9)	C55—Zr3—C54	30.54 (11)
C14—Zr1—C13	31.15 (9)	C52—C51—C55	108.4 (3)
C11—Zr1—C4	148.27 (10)	C52—C51—Zr3	74.22 (18)
C15—Zr1—C4	119.31 (9)	C55—C51—Zr3	75.18 (18)
C12—Zr1—C4	165.27 (10)	C52—C51—H51	125.8
C3—Zr1—C4	31.43 (9)	C55—C51—H51	125.8
C1—Zr1—C4	51.37 (10)	Zr3—C51—H51	116.8
C9—Zr1—C4	100.62 (10)	C51—C52—C53	108.6 (3)
C16—Zr1—C4	130.95 (10)	C51—C52—Zr3	75.44 (17)
C6—Zr1—C4	95.81 (9)	C53—C52—Zr3	72.53 (16)
C5—Zr1—C4	30.58 (10)	C51—C52—H52	125.7
C10—Zr1—C4	117.07 (9)	C53—C52—H52	125.7
C8—Zr1—C4	69.97 (10)	Zr3—C52—H52	118.2
C2—Zr1—C4	51.17 (9)	C52—C53—C54	107.5 (3)
C14—Zr1—C4	114.13 (9)	C52—C53—Zr3	76.09 (17)
C13—Zr1—C4	133.51 (9)	C54—C53—Zr3	77.70 (17)
C11—Zr1—C7	137.70 (9)	C52—C53—H53	126.3
C15—Zr1—C7	172.12 (9)	C54—C53—H53	126.3
C12—Zr1—C7	120.17 (9)	Zr3—C53—H53	112.5
C3—Zr1—C7	95.22 (9)	C55—C54—C53	108.0 (3)
C1—Zr1—C7	102.75 (9)	C55—C54—Zr3	74.56 (17)
C9—Zr1—C7	51.36 (9)	C53—C54—Zr3	71.16 (16)
C16—Zr1—C7	113.32 (9)	C55—C54—H54	126.0
C6—Zr1—C7	31.10 (9)	C53—C54—H54	126.0
C5—Zr1—C7	71.72 (9)	Zr3—C54—H54	120.1
C10—Zr1—C7	51.17 (9)	C54—C55—C51	107.3 (3)
C8—Zr1—C7	30.76 (10)	C54—C55—Zr3	74.90 (18)
C2—Zr1—C7	117.66 (9)	C51—C55—Zr3	73.74 (19)
C14—Zr1—C7	152.20 (10)	C54—C55—H55	126.3
C13—Zr1—C7	126.86 (9)	C51—C55—H55	126.3
C4—Zr1—C7	67.17 (9)	Zr3—C55—H55	117.1
C2—C1—C5	107.4 (3)	C60—C56—C57	106.8 (3)
C2—C1—Zr1	75.22 (18)	C60—C56—Zr3	75.73 (17)
C5—C1—Zr1	74.78 (18)	C57—C56—Zr3	75.00 (16)
C2—C1—H1	126.3	C60—C56—H56	126.6
C5—C1—H1	126.3	C57—C56—H56	126.6
Zr1—C1—H1	115.9	Zr3—C56—H56	115.1
C1—C2—C3	108.5 (3)	C58—C57—C56	107.4 (3)
C1—C2—Zr1	73.73 (17)	C58—C57—Zr3	74.91 (15)
C3—C2—Zr1	72.64 (16)	C56—C57—Zr3	73.34 (15)
C1—C2—H2	125.8	C58—C57—H57	126.3
C3—C2—H2	125.8	C56—C57—H57	126.3
Zr1—C2—H2	119.7	Zr3—C57—H57	117.5
C2—C3—C4	107.0 (3)	C59—C58—C57	109.5 (3)
C2—C3—Zr1	75.91 (17)	C59—C58—Zr3	73.97 (16)
C4—C3—Zr1	76.36 (16)	C57—C58—Zr3	74.45 (15)
C2—C3—H3	126.5	C59—C58—H58	125.3
C4—C3—H3	126.5	C57—C58—H58	125.3
Zr1—C3—H3	113.8	Zr3—C58—H58	118.1
C5—C4—C3	108.6 (3)	C58—C59—C60	108.3 (3)
C5—C4—Zr1	74.04 (17)	C58—C59—Zr3	75.48 (17)
C3—C4—Zr1	72.21 (15)	C60—C59—Zr3	75.68 (18)
C5—C4—H4	125.7	C58—C59—H59	125.9
C3—C4—H4	125.7	C60—C59—H59	125.9
Zr1—C4—H4	119.8	Zr3—C59—H59	115.2
C4—C5—C1	108.3 (3)	C59—C60—C56	107.9 (3)
C4—C5—Zr1	75.38 (17)	C59—C60—Zr3	73.47 (17)
C1—C5—Zr1	73.72 (17)	C56—C60—Zr3	72.90 (17)
C4—C5—H5	125.9	C59—C60—H60	126.0
C1—C5—H5	125.9	C56—C60—H60	126.0
Zr1—C5—H5	117.0	Zr3—C60—H60	119.5
C10—C6—C7	107.4 (3)	C62—C61—C65	104.9 (3)
C10—C6—Zr1	74.42 (15)	C62—C61—C66	123.3 (2)
C7—C6—Zr1	76.01 (15)	C65—C61—C66	122.7 (3)
C10—C6—H6	126.3	C62—C61—Zr3	78.99 (15)
C7—C6—H6	126.3	C65—C61—Zr3	77.91 (15)
Zr1—C6—H6	115.5	C66—C61—Zr3	82.49 (15)
C8—C7—C6	108.1 (3)	C63—C62—C61	109.3 (3)
C8—C7—Zr1	73.28 (15)	C63—C62—Zr3	79.71 (17)
C6—C7—Zr1	72.89 (14)	C61—C62—Zr3	66.55 (14)
C8—C7—H7	126.0	C63—C62—H62	125.3
C6—C7—H7	126.0	C61—C62—H62	125.3
Zr1—C7—H7	119.7	Zr3—C62—H62	119.9
C7—C8—C9	108.3 (3)	C62—C63—C64	108.3 (3)
C7—C8—Zr1	75.97 (16)	C62—C63—Zr3	68.77 (16)
C9—C8—Zr1	73.71 (16)	C64—C63—Zr3	73.64 (17)
C7—C8—H8	125.8	C62—C63—H63	125.9
C9—C8—H8	125.8	C64—C63—H63	125.9
Zr1—C8—H8	116.5	Zr3—C63—H63	123.3
C10—C9—C8	107.6 (3)	C65—C64—C63	107.8 (3)
C10—C9—Zr1	74.90 (17)	C65—C64—Zr3	68.40 (15)
C8—C9—Zr1	74.91 (17)	C63—C64—Zr3	75.05 (16)
C10—C9—H9	126.2	C65—C64—H64	126.1
C8—C9—H9	126.2	C63—C64—H64	126.1
Zr1—C9—H9	116.2	Zr3—C64—H64	122.1
C9—C10—C6	108.5 (3)	C64—C65—C61	109.4 (3)
C9—C10—Zr1	73.99 (16)	C64—C65—Zr3	79.75 (15)
C6—C10—Zr1	74.27 (16)	C61—C65—Zr3	67.32 (13)
C9—C10—H10	125.8	C64—C65—H65	125.3
C6—C10—H10	125.8	C61—C65—H65	125.3
Zr1—C10—H10	117.9	Zr3—C65—H65	119.2
C15—C11—C12	105.3 (2)	C61—C66—C71	118.2 (3)
C15—C11—C16	121.7 (2)	C61—C66—C67	117.3 (2)
C12—C11—C16	124.6 (2)	C71—C66—C67	109.4 (3)
C15—C11—Zr1	78.38 (14)	C61—C66—Zr3	63.80 (14)
C12—C11—Zr1	78.57 (14)	C71—C66—Zr3	119.42 (18)
C16—C11—Zr1	83.55 (15)	C67—C66—Zr3	122.33 (19)
C13—C12—C11	109.0 (2)	C66—C67—C68	111.0 (3)
C13—C12—Zr1	79.43 (15)	C66—C67—C72	108.5 (3)
C11—C12—Zr1	66.92 (13)	C68—C67—C72	108.7 (3)
C13—C12—H12	125.5	C66—C67—H67	109.6
C11—C12—H12	125.5	C68—C67—H67	109.6
Zr1—C12—H12	119.7	C72—C67—H67	109.6
C12—C13—C14	108.2 (3)	C69—C68—C67	110.0 (3)
C12—C13—Zr1	68.78 (14)	C69—C68—H68A	109.7
C14—C13—Zr1	74.31 (15)	C67—C68—H68A	109.7
C12—C13—H13	125.9	C69—C68—H68B	109.7
C14—C13—H13	125.9	C67—C68—H68B	109.7
Zr1—C13—H13	122.6	H68A—C68—H68B	108.2
C15—C14—C13	108.1 (2)	C68—C69—C70	109.2 (3)
C15—C14—Zr1	68.68 (13)	C68—C69—C73	109.4 (3)
C13—C14—Zr1	74.54 (14)	C70—C69—C73	109.4 (3)
C15—C14—H14	126.0	C68—C69—H69	109.6
C13—C14—H14	126.0	C70—C69—H69	109.6
Zr1—C14—H14	122.5	C73—C69—H69	109.6
C14—C15—C11	109.1 (2)	C69—C70—C71	110.3 (3)
C14—C15—Zr1	79.47 (14)	C69—C70—H70A	109.6
C11—C15—Zr1	67.08 (13)	C71—C70—H70A	109.6
C14—C15—H15	125.5	C69—C70—H70B	109.6
C11—C15—H15	125.5	C71—C70—H70B	109.6
Zr1—C15—H15	119.5	H70A—C70—H70B	108.1
C11—C16—C17	116.9 (2)	C66—C71—C70	110.4 (3)
C11—C16—C21	118.7 (3)	C66—C71—C74	108.5 (2)
C17—C16—C21	109.4 (2)	C70—C71—C74	108.6 (3)
C11—C16—Zr1	62.92 (14)	C66—C71—H71	109.8
C17—C16—Zr1	123.48 (19)	C70—C71—H71	109.8
C21—C16—Zr1	118.71 (18)	C74—C71—H71	109.8
C16—C17—C18	111.1 (2)	C75—C72—C67	109.3 (3)
C16—C17—C22	108.3 (2)	C75—C72—H72A	109.8
C18—C17—C22	108.6 (3)	C67—C72—H72A	109.8
C16—C17—H17	109.6	C75—C72—H72B	109.8
C18—C17—H17	109.6	C67—C72—H72B	109.8
C22—C17—H17	109.6	H72A—C72—H72B	108.3
C19—C18—C17	109.8 (3)	C75—C73—C69	109.4 (3)
C19—C18—H18A	109.7	C75—C73—H73A	109.8
C17—C18—H18A	109.7	C69—C73—H73A	109.8
C19—C18—H18B	109.7	C75—C73—H73B	109.8
C17—C18—H18B	109.7	C69—C73—H73B	109.8
H18A—C18—H18B	108.2	H73A—C73—H73B	108.2
C23—C19—C18	110.3 (3)	C75—C74—C71	109.9 (3)
C23—C19—C20	109.7 (3)	C75—C74—H74A	109.7
C18—C19—C20	108.1 (3)	C71—C74—H74A	109.7
C23—C19—H19	109.6	C75—C74—H74B	109.7
C18—C19—H19	109.6	C71—C74—H74B	109.7
C20—C19—H19	109.6	H74A—C74—H74B	108.2
C21—C20—C19	110.1 (2)	C73—C75—C74	110.1 (3)
C21—C20—H20A	109.6	C73—C75—C72	109.5 (3)
C19—C20—H20A	109.6	C74—C75—C72	108.9 (3)
C21—C20—H20B	109.6	C73—C75—H75	109.5
C19—C20—H20B	109.6	C74—C75—H75	109.5
H20A—C20—H20B	108.2	C72—C75—H75	109.5
C16—C21—C20	110.5 (3)	C86—Zr4—C87	34.75 (9)
C16—C21—C24	108.8 (2)	C86—Zr4—C90	34.31 (10)
C20—C21—C24	108.1 (3)	C87—Zr4—C90	54.72 (9)
C16—C21—H21	109.8	C86—Zr4—C83	132.39 (9)
C20—C21—H21	109.8	C87—Zr4—C83	98.55 (9)
C24—C21—H21	109.8	C90—Zr4—C83	142.17 (9)
C25—C22—C17	110.0 (2)	C86—Zr4—C91	33.84 (9)
C25—C22—H22A	109.7	C87—Zr4—C91	60.07 (9)
C17—C22—H22A	109.7	C90—Zr4—C91	59.99 (10)
C25—C22—H22B	109.7	C83—Zr4—C91	134.28 (10)
C17—C22—H22B	109.7	C86—Zr4—C78	118.07 (10)
H22A—C22—H22B	108.2	C87—Zr4—C78	130.64 (9)
C19—C23—C25	109.5 (3)	C90—Zr4—C78	84.54 (10)
C19—C23—H23A	109.8	C83—Zr4—C78	99.37 (10)
C25—C23—H23A	109.8	C91—Zr4—C78	125.61 (10)
C19—C23—H23B	109.8	C86—Zr4—C76	102.33 (9)
C25—C23—H23B	109.8	C87—Zr4—C76	136.89 (10)
H23A—C23—H23B	108.2	C90—Zr4—C76	87.52 (9)
C25—C24—C21	109.9 (3)	C83—Zr4—C76	124.43 (9)
C25—C24—H24A	109.7	C91—Zr4—C76	84.50 (10)
C21—C24—H24A	109.7	C78—Zr4—C76	51.81 (11)
C25—C24—H24B	109.7	C86—Zr4—C88	55.31 (10)
C21—C24—H24B	109.7	C87—Zr4—C88	31.78 (9)
H24A—C24—H24B	108.2	C90—Zr4—C88	52.75 (9)
C24—C25—C23	109.6 (3)	C83—Zr4—C88	90.09 (9)
C24—C25—C22	109.6 (3)	C91—Zr4—C88	87.66 (9)
C23—C25—C22	108.9 (3)	C78—Zr4—C88	102.87 (10)
C24—C25—H25	109.6	C76—Zr4—C88	137.11 (10)
C23—C25—H25	109.6	C86—Zr4—C81	116.39 (10)
C22—C25—H25	109.6	C87—Zr4—C81	104.40 (10)
C36—Zr2—C40	34.76 (9)	C90—Zr4—C81	150.47 (10)
C36—Zr2—C37	34.37 (9)	C83—Zr4—C81	51.73 (10)
C40—Zr2—C37	54.85 (9)	C91—Zr4—C81	92.22 (10)
C36—Zr2—C34	112.54 (13)	C78—Zr4—C81	122.70 (10)
C40—Zr2—C34	127.93 (12)	C76—Zr4—C81	100.41 (10)
C37—Zr2—C34	79.37 (12)	C88—Zr4—C81	122.02 (10)
C36—Zr2—C33	143.00 (11)	C86—Zr4—C77	95.22 (9)
C40—Zr2—C33	153.28 (14)	C87—Zr4—C77	122.33 (9)
C37—Zr2—C33	110.60 (12)	C90—Zr4—C77	67.66 (9)
C34—Zr2—C33	31.23 (13)	C83—Zr4—C77	128.99 (10)
C36—Zr2—C28	124.58 (10)	C91—Zr4—C77	94.62 (10)
C40—Zr2—C28	90.57 (10)	C78—Zr4—C77	31.22 (11)
C37—Zr2—C28	135.72 (10)	C76—Zr4—C77	31.12 (11)
C34—Zr2—C28	110.53 (14)	C88—Zr4—C77	108.44 (10)
C33—Zr2—C28	87.93 (11)	C81—Zr4—C77	129.29 (10)
C36—Zr2—C26	98.65 (10)	C86—Zr4—C79	146.40 (10)
C40—Zr2—C26	81.99 (12)	C87—Zr4—C79	158.15 (10)
C37—Zr2—C26	132.45 (11)	C90—Zr4—C79	115.10 (9)
C34—Zr2—C26	148.09 (14)	C83—Zr4—C79	79.20 (9)
C33—Zr2—C26	116.88 (14)	C91—Zr4—C79	135.54 (9)
C28—Zr2—C26	51.39 (10)	C78—Zr4—C79	31.35 (9)
C36—Zr2—C39	55.56 (9)	C76—Zr4—C79	51.33 (10)
C40—Zr2—C39	31.98 (9)	C88—Zr4—C79	126.45 (10)
C37—Zr2—C39	53.04 (9)	C81—Zr4—C79	91.35 (10)
C34—Zr2—C39	102.03 (12)	C77—Zr4—C79	51.26 (9)
C33—Zr2—C39	121.60 (13)	C86—Zr4—C84	101.91 (9)
C28—Zr2—C39	82.82 (10)	C87—Zr4—C84	70.22 (9)
C26—Zr2—C39	101.06 (12)	C90—Zr4—C84	122.99 (9)
C36—Zr2—C41	33.30 (9)	C83—Zr4—C84	31.40 (9)
C40—Zr2—C41	59.52 (9)	C91—Zr4—C84	105.97 (10)
C37—Zr2—C41	59.62 (8)	C78—Zr4—C84	128.28 (11)
C34—Zr2—C41	119.76 (13)	C76—Zr4—C84	149.17 (9)
C33—Zr2—C41	137.08 (10)	C88—Zr4—C84	73.21 (9)
C28—Zr2—C41	129.71 (9)	C81—Zr4—C84	51.25 (10)
C26—Zr2—C41	82.96 (9)	C77—Zr4—C84	159.41 (11)
C39—Zr2—C41	87.35 (9)	C79—Zr4—C84	110.55 (9)
C36—Zr2—C35	94.17 (10)	C86—Zr4—C82	144.70 (10)
C40—Zr2—C35	123.59 (9)	C87—Zr4—C82	120.51 (9)
C37—Zr2—C35	68.87 (10)	C90—Zr4—C82	173.47 (9)
C34—Zr2—C35	30.77 (14)	C83—Zr4—C82	31.36 (9)
C33—Zr2—C35	50.69 (11)	C91—Zr4—C82	122.81 (10)
C28—Zr2—C35	138.38 (11)	C78—Zr4—C82	97.21 (10)
C26—Zr2—C35	145.22 (14)	C76—Zr4—C82	98.52 (9)
C39—Zr2—C35	112.79 (11)	C88—Zr4—C82	120.75 (9)
C41—Zr2—C35	90.63 (11)	C81—Zr4—C82	30.78 (10)
C36—Zr2—C31	106.40 (11)	C77—Zr4—C82	116.71 (9)
C40—Zr2—C31	141.15 (10)	C79—Zr4—C82	67.60 (9)
C37—Zr2—C31	92.85 (11)	C84—Zr4—C82	51.24 (8)
C34—Zr2—C31	51.07 (13)	C86—Zr4—C80	132.47 (9)
C33—Zr2—C31	50.71 (12)	C87—Zr4—C80	166.93 (10)
C28—Zr2—C31	127.68 (11)	C90—Zr4—C80	117.13 (9)
C26—Zr2—C31	114.70 (13)	C83—Zr4—C80	93.28 (9)
C39—Zr2—C31	142.67 (11)	C91—Zr4—C80	107.43 (9)
C41—Zr2—C31	86.75 (10)	C78—Zr4—C80	51.76 (10)
C35—Zr2—C31	30.57 (13)	C76—Zr4—C80	31.21 (9)
C36—Zr2—C30	126.34 (12)	C88—Zr4—C80	154.62 (10)
C40—Zr2—C30	112.79 (11)	C81—Zr4—C80	78.66 (10)
C37—Zr2—C30	160.27 (12)	C77—Zr4—C80	51.40 (9)
C34—Zr2—C30	117.50 (14)	C79—Zr4—C80	30.90 (10)
C33—Zr2—C30	86.64 (15)	C84—Zr4—C80	119.65 (9)
C28—Zr2—C30	51.07 (11)	C82—Zr4—C80	68.43 (9)
C26—Zr2—C30	30.99 (12)	C86—Zr4—C85	93.83 (10)
C39—Zr2—C30	126.36 (11)	C87—Zr4—C85	73.93 (9)
C41—Zr2—C30	101.33 (12)	C90—Zr4—C85	126.30 (9)
C35—Zr2—C30	119.82 (12)	C83—Zr4—C85	51.63 (10)
C31—Zr2—C30	90.93 (12)	C91—Zr4—C85	82.80 (10)
C36—Zr2—C38	55.18 (9)	C78—Zr4—C85	147.88 (10)
C40—Zr2—C38	53.00 (9)	C76—Zr4—C85	128.56 (11)
C37—Zr2—C38	31.69 (9)	C88—Zr4—C85	91.87 (10)
C34—Zr2—C38	75.14 (11)	C81—Zr4—C85	31.19 (10)
C33—Zr2—C38	102.14 (13)	C77—Zr4—C85	159.46 (11)
C28—Zr2—C38	106.89 (10)	C79—Zr4—C85	118.55 (9)
C26—Zr2—C38	131.84 (12)	C84—Zr4—C85	30.72 (10)
C39—Zr2—C38	31.24 (9)	C82—Zr4—C85	51.11 (9)
C41—Zr2—C38	87.14 (8)	C80—Zr4—C85	109.85 (10)
C35—Zr2—C38	81.55 (11)	C86—Zr4—C89	54.90 (10)
C31—Zr2—C38	111.60 (11)	C87—Zr4—C89	52.66 (9)
C30—Zr2—C38	156.48 (10)	C90—Zr4—C89	31.52 (10)
C36—Zr2—C27	97.99 (9)	C83—Zr4—C89	112.14 (9)
C40—Zr2—C27	68.36 (9)	C91—Zr4—C89	87.38 (9)
C37—Zr2—C27	122.77 (9)	C78—Zr4—C89	77.98 (9)
C34—Zr2—C27	141.41 (14)	C76—Zr4—C89	106.43 (10)
C33—Zr2—C27	117.66 (11)	C88—Zr4—C89	30.96 (9)
C28—Zr2—C27	31.10 (10)	C81—Zr4—C89	152.98 (9)
C26—Zr2—C27	30.68 (11)	C77—Zr4—C89	77.60 (9)
C39—Zr2—C27	75.71 (10)	C79—Zr4—C89	107.72 (9)
C41—Zr2—C27	98.76 (9)	C84—Zr4—C89	103.01 (9)
C35—Zr2—C27	167.76 (10)	C82—Zr4—C89	142.69 (10)
C31—Zr2—C27	141.62 (12)	C80—Zr4—C89	127.12 (10)
C30—Zr2—C27	50.70 (11)	C85—Zr4—C89	122.44 (10)
C38—Zr2—C27	106.61 (10)	C77—C76—C80	108.3 (3)
C36—Zr2—C29	147.40 (10)	C77—C76—Zr4	75.14 (16)
C40—Zr2—C29	118.25 (10)	C80—C76—Zr4	75.47 (15)
C37—Zr2—C29	165.16 (12)	C77—C76—H76	125.8
C34—Zr2—C29	99.56 (14)	C80—C76—H76	125.8
C33—Zr2—C29	69.59 (12)	Zr4—C76—H76	115.7
C28—Zr2—C29	30.84 (11)	C76—C77—C78	108.1 (3)
C26—Zr2—C29	50.63 (11)	C76—C77—Zr4	73.74 (17)
C39—Zr2—C29	113.48 (11)	C78—C77—Zr4	73.26 (17)
C41—Zr2—C29	130.91 (11)	C76—C77—H77	126.0
C35—Zr2—C29	117.27 (10)	C78—C77—H77	126.0
C31—Zr2—C29	97.94 (12)	Zr4—C77—H77	118.9
C30—Zr2—C29	30.30 (12)	C77—C78—C79	107.5 (3)
C38—Zr2—C29	133.58 (12)	C77—C78—Zr4	75.53 (18)
C27—Zr2—C29	50.49 (10)	C79—C78—Zr4	75.56 (17)
C36—Zr2—C32	136.79 (11)	C77—C78—H78	126.2
C40—Zr2—C32	171.54 (11)	C79—C78—H78	126.2
C37—Zr2—C32	118.85 (10)	Zr4—C78—H78	115.0
C34—Zr2—C32	50.65 (13)	C80—C79—C78	108.5 (3)
C33—Zr2—C32	30.26 (14)	C80—C79—Zr4	74.83 (16)
C28—Zr2—C32	97.63 (11)	C78—C79—Zr4	73.09 (16)
C26—Zr2—C32	101.53 (13)	C80—C79—H79	125.7
C39—Zr2—C32	151.19 (12)	C78—C79—H79	125.7
C41—Zr2—C32	112.99 (12)	Zr4—C79—H79	118.2
C35—Zr2—C32	50.05 (10)	C79—C80—C76	107.5 (3)
C31—Zr2—C32	30.43 (11)	C79—C80—Zr4	74.28 (15)
C30—Zr2—C32	71.45 (12)	C76—C80—Zr4	73.32 (14)
C38—Zr2—C32	125.53 (11)	C79—C80—H80	126.3
C27—Zr2—C32	118.36 (10)	C76—C80—H80	126.3
C29—Zr2—C32	69.27 (11)	Zr4—C80—H80	118.1
C27—C26—C30	107.8 (3)	C82—C81—C85	108.0 (3)
C27—C26—Zr2	75.73 (17)	C82—C81—Zr4	74.95 (17)
C30—C26—Zr2	74.96 (18)	C85—C81—Zr4	74.82 (18)
C27—C26—H26	126.1	C82—C81—H81	126.0
C30—C26—H26	126.1	C85—C81—H81	126.0
Zr2—C26—H26	115.4	Zr4—C81—H81	116.3
C26—C27—C28	108.1 (3)	C81—C82—C83	108.0 (3)
C26—C27—Zr2	73.59 (17)	C81—C82—Zr4	74.27 (16)
C28—C27—Zr2	73.35 (16)	C83—C82—Zr4	71.97 (15)
C26—C27—H27	125.9	C81—C82—H82	126.0
C28—C27—H27	125.9	C83—C82—H82	126.0
Zr2—C27—H27	119.0	Zr4—C82—H82	119.6
C29—C28—C27	106.9 (3)	C82—C83—C84	107.8 (3)
C29—C28—Zr2	75.84 (17)	C82—C83—Zr4	76.66 (16)
C27—C28—Zr2	75.55 (16)	C84—C83—Zr4	76.52 (16)
C29—C28—H28	126.6	C82—C83—H83	126.1
C27—C28—H28	126.6	C84—C83—H83	126.1
Zr2—C28—H28	114.5	Zr4—C83—H83	113.2
C30—C29—C28	108.9 (3)	C85—C84—C83	108.0 (3)
C30—C29—Zr2	74.08 (17)	C85—C84—Zr4	74.84 (17)
C28—C29—Zr2	73.31 (16)	C83—C84—Zr4	72.08 (16)
C30—C29—H29	125.6	C85—C84—H84	126.0
C28—C29—H29	125.6	C83—C84—H84	126.0
Zr2—C29—H29	118.9	Zr4—C84—H84	119.0
C29—C30—C26	108.1 (3)	C84—C85—C81	108.0 (3)
C29—C30—Zr2	75.62 (18)	C84—C85—Zr4	74.44 (18)
C26—C30—Zr2	74.04 (17)	C81—C85—Zr4	73.99 (18)
C29—C30—H30	125.9	C84—C85—H85	126.0
C26—C30—H30	125.9	C81—C85—H85	126.0
Zr2—C30—H30	116.5	Zr4—C85—H85	117.6
C35—C31—C32	107.4 (4)	C90—C86—C87	105.3 (3)
C35—C31—Zr2	74.7 (2)	C90—C86—C91	123.6 (2)
C32—C31—Zr2	75.9 (2)	C87—C86—C91	122.2 (3)
C35—C31—H31	126.3	C90—C86—Zr4	79.46 (15)
C32—C31—H31	126.3	C87—C86—Zr4	77.95 (15)
Zr2—C31—H31	115.4	C91—C86—Zr4	82.42 (15)
C33—C32—C31	108.7 (3)	C88—C87—C86	109.0 (3)
C33—C32—Zr2	73.2 (2)	C88—C87—Zr4	80.04 (15)
C31—C32—Zr2	73.68 (19)	C86—C87—Zr4	67.31 (13)
C33—C32—H32	125.7	C88—C87—H87	125.5
C31—C32—H32	125.7	C86—C87—H87	125.5
Zr2—C32—H32	119.2	Zr4—C87—H87	118.7
C32—C33—C34	107.8 (3)	C87—C88—C89	108.2 (3)
C32—C33—Zr2	76.5 (2)	C87—C88—Zr4	68.18 (14)
C34—C33—Zr2	73.3 (2)	C89—C88—Zr4	75.48 (15)
C32—C33—H33	126.1	C87—C88—H88	125.9
C34—C33—H33	126.1	C89—C88—H88	125.9
Zr2—C33—H33	116.2	Zr4—C88—H88	122.1
C35—C34—C33	107.4 (4)	C90—C89—C88	108.3 (3)
C35—C34—Zr2	76.2 (2)	C90—C89—Zr4	68.67 (15)
C33—C34—Zr2	75.4 (2)	C88—C89—Zr4	73.56 (15)
C35—C34—H34	126.3	C90—C89—H89	125.8
C33—C34—H34	126.3	C88—C89—H89	125.8
Zr2—C34—H34	114.5	Zr4—C89—H89	123.5
C34—C35—C31	108.6 (3)	C89—C90—C86	108.9 (2)
C34—C35—Zr2	73.1 (2)	C89—C90—Zr4	79.80 (16)
C31—C35—Zr2	74.76 (19)	C86—C90—Zr4	66.23 (14)
C34—C35—H35	125.7	C89—C90—H90	125.6
C31—C35—H35	125.7	C86—C90—H90	125.6
Zr2—C35—H35	118.3	Zr4—C90—H90	119.9
C37—C36—C41	124.0 (3)	C86—C91—C96	116.4 (2)
C37—C36—C40	105.3 (2)	C86—C91—C92	118.5 (3)
C41—C36—C40	122.2 (2)	C96—C91—C92	109.5 (3)
C37—C36—Zr2	78.98 (14)	C86—C91—Zr4	63.74 (15)
C41—C36—Zr2	83.75 (15)	C96—C91—Zr4	123.97 (19)
C40—C36—Zr2	77.67 (14)	C92—C91—Zr4	118.07 (19)
C38—C37—C36	109.0 (2)	C91—C92—C93	110.1 (3)
C38—C37—Zr2	79.23 (14)	C91—C92—C97	108.7 (2)
C36—C37—Zr2	66.65 (13)	C93—C92—C97	108.3 (3)
C38—C37—H37	125.5	C91—C92—H92	109.9
C36—C37—H37	125.5	C93—C92—H92	109.9
Zr2—C37—H37	120.1	C97—C92—H92	109.9
C37—C38—C39	108.3 (3)	C94—C93—C92	110.6 (3)
C37—C38—Zr2	69.08 (14)	C94—C93—H93A	109.5
C39—C38—Zr2	73.52 (15)	C92—C93—H93A	109.5
C37—C38—H38	125.9	C94—C93—H93B	109.5
C39—C38—H38	125.9	C92—C93—H93B	109.5
Zr2—C38—H38	123.2	H93A—C93—H93B	108.1
C40—C39—C38	108.2 (3)	C95—C94—C93	108.7 (3)
C40—C39—Zr2	68.67 (15)	C95—C94—C98	109.7 (3)
C38—C39—Zr2	75.23 (16)	C93—C94—C98	109.4 (3)
C40—C39—H39	125.9	C95—C94—H94	109.6
C38—C39—H39	125.9	C93—C94—H94	109.6
Zr2—C39—H39	121.8	C98—C94—H94	109.6
C39—C40—C36	108.9 (2)	C94—C95—C96	110.2 (3)
C39—C40—Zr2	79.35 (16)	C94—C95—H95A	109.6
C36—C40—Zr2	67.57 (14)	C96—C95—H95A	109.6
C39—C40—H40	125.5	C94—C95—H95B	109.6
C36—C40—H40	125.5	C96—C95—H95B	109.6
Zr2—C40—H40	119.2	H95A—C95—H95B	108.1
C36—C41—C46	118.4 (2)	C95—C96—C91	111.0 (3)
C36—C41—C42	116.8 (2)	C95—C96—C99	108.7 (3)
C46—C41—C42	109.7 (2)	C91—C96—C99	108.3 (3)
C36—C41—Zr2	62.95 (14)	C95—C96—H96	109.6
C46—C41—Zr2	120.21 (18)	C91—C96—H96	109.6
C42—C41—Zr2	121.86 (16)	C99—C96—H96	109.6
C43—C42—C41	111.0 (3)	C100—C97—C92	109.8 (3)
C43—C42—C47	108.4 (3)	C100—C97—H97A	109.7
C41—C42—C47	108.7 (2)	C92—C97—H97A	109.7
C43—C42—H42	109.6	C100—C97—H97B	109.7
C41—C42—H42	109.6	C92—C97—H97B	109.7
C47—C42—H42	109.6	H97A—C97—H97B	108.2
C42—C43—C44	109.7 (2)	C100—C98—C94	109.2 (3)
C42—C43—H43A	109.7	C100—C98—H98A	109.8
C44—C43—H43A	109.7	C94—C98—H98A	109.8
C42—C43—H43B	109.7	C100—C98—H98B	109.8
C44—C43—H43B	109.7	C94—C98—H98B	109.8
H43A—C43—H43B	108.2	H98A—C98—H98B	108.3
C48—C44—C43	110.2 (3)	C100—C99—C96	109.6 (3)
C48—C44—C45	109.7 (3)	C100—C99—H99A	109.7
C43—C44—C45	108.7 (3)	C96—C99—H99A	109.7
C48—C44—H44	109.4	C100—C99—H99B	109.7
C43—C44—H44	109.4	C96—C99—H99B	109.7
C45—C44—H44	109.4	H99A—C99—H99B	108.2
C46—C45—C44	109.9 (3)	C98—C100—C97	109.8 (3)
C46—C45—H45A	109.7	C98—C100—C99	109.4 (3)
C44—C45—H45A	109.7	C97—C100—C99	109.4 (3)
C46—C45—H45B	109.7	C98—C100—H100	109.4
C44—C45—H45B	109.7	C97—C100—H100	109.4
H45A—C45—H45B	108.2	C99—C100—H100	109.4
C41—C46—C45	110.7 (2)	C106—C101—C107	109.2 (14)
C41—C46—C49	108.4 (2)	C106—C101—C102	123.9 (14)
C45—C46—C49	108.4 (3)	C107—C101—C102	126.8 (17)
C41—C46—H46	109.8	C103—C102—C101	119.8 (10)
C45—C46—H46	109.8	C103—C102—H102	120.1
C49—C46—H46	109.8	C101—C102—H102	120.1
C50—C47—C42	109.7 (3)	C102—C103—C104	122.4 (11)
C50—C47—H47A	109.7	C102—C103—H103	118.8
C42—C47—H47A	109.7	C104—C103—H103	118.8
C50—C47—H47B	109.7	C103—C104—C105	115.0 (14)
C42—C47—H47B	109.7	C103—C104—H104	122.5
H47A—C47—H47B	108.2	C105—C104—H104	122.5
C50—C48—C44	109.4 (3)	C106—C105—C104	123.4 (11)
C50—C48—H48A	109.8	C106—C105—H105	118.3
C44—C48—H48A	109.8	C104—C105—H105	118.3
C50—C48—H48B	109.8	C105—C106—C101	115.1 (11)
C44—C48—H48B	109.8	C105—C106—H106	122.4
H48A—C48—H48B	108.2	C101—C106—H106	122.4
C50—C49—C46	109.6 (2)	C101—C107—H10F	109.5
C50—C49—H49A	109.7	C101—C107—H10G	109.5
C46—C49—H49A	109.7	H10F—C107—H10G	109.5
C50—C49—H49B	109.7	C101—C107—H10H	109.5
C46—C49—H49B	109.7	H10F—C107—H10H	109.5
H49A—C49—H49B	108.2	H10G—C107—H10H	109.5
C48—C50—C47	109.5 (3)	C109—C108—H10A	109.5
C48—C50—C49	109.2 (3)	C109—C108—H10B	109.5
C47—C50—C49	109.4 (3)	H10A—C108—H10B	109.5
C48—C50—H50	109.6	C109—C108—H10C	109.5
C47—C50—H50	109.6	H10A—C108—H10C	109.5
C49—C50—H50	109.6	H10B—C108—H10C	109.5
C61—Zr3—C65	34.77 (10)	C110—C109—C108	109.6 (13)
C61—Zr3—C62	34.46 (10)	C110—C109—H10D	109.7
C65—Zr3—C62	54.74 (9)	C108—C109—H10D	109.7
C61—Zr3—C53	129.88 (9)	C110—C109—H10E	109.7
C65—Zr3—C53	96.00 (9)	C108—C109—H10E	109.7
C62—Zr3—C53	140.09 (10)	H10D—C109—H10E	108.2
C61—Zr3—C66	33.70 (10)	C111—C110—C109	112.4 (15)
C65—Zr3—C66	60.12 (10)	C111—C110—H11A	109.1
C62—Zr3—C66	59.95 (10)	C109—C110—H11A	109.1
C53—Zr3—C66	133.08 (9)	C111—C110—H11B	109.1
C61—Zr3—C56	102.48 (10)	C109—C110—H11B	109.1
C65—Zr3—C56	137.01 (10)	H11A—C110—H11B	107.9
C62—Zr3—C56	87.42 (11)	C110—C111—C112	102.9 (14)
C53—Zr3—C56	126.91 (10)	C110—C111—H11C	111.2
C66—Zr3—C56	84.74 (10)	C112—C111—H11C	111.2
C61—Zr3—C59	119.11 (11)	C110—C111—H11D	111.2
C65—Zr3—C59	131.17 (11)	C112—C111—H11D	111.2
C62—Zr3—C59	85.40 (10)	H11C—C111—H11D	109.1
C53—Zr3—C59	100.06 (11)	C113—C112—C111	107.4 (11)
C66—Zr3—C59	126.39 (11)	C113—C112—H11E	110.2
C56—Zr3—C59	51.76 (12)	C111—C112—H11E	110.2
C61—Zr3—C57	133.05 (10)	C113—C112—H11F	110.2
C65—Zr3—C57	167.57 (10)	C111—C112—H11F	110.2
C62—Zr3—C57	117.43 (9)	H11E—C112—H11F	108.5
C53—Zr3—C57	95.31 (10)	C112—C113—H11G	109.5
C66—Zr3—C57	108.03 (10)	C112—C113—H11H	109.5
C56—Zr3—C57	31.66 (10)	H11G—C113—H11H	109.5
C59—Zr3—C57	51.17 (11)	C112—C113—H11I	109.5
C61—Zr3—C52	100.23 (9)	H11G—C113—H11I	109.5
C65—Zr3—C52	69.21 (9)	H11H—C113—H11I	109.5
			
C5—C1—C2—C3	3.6 (3)	C52—C53—C54—C55	4.9 (3)
Zr1—C1—C2—C3	−64.8 (2)	Zr3—C53—C54—C55	−66.0 (2)
C5—C1—C2—Zr1	68.4 (2)	C52—C53—C54—Zr3	70.9 (2)
C1—C2—C3—C4	−5.1 (3)	C53—C54—C55—C51	−3.4 (3)
Zr1—C2—C3—C4	−70.7 (2)	Zr3—C54—C55—C51	−67.2 (2)
C1—C2—C3—Zr1	65.6 (2)	C53—C54—C55—Zr3	63.8 (2)
C2—C3—C4—C5	4.7 (3)	C52—C51—C55—C54	0.5 (4)
Zr1—C3—C4—C5	−65.6 (2)	Zr3—C51—C55—C54	67.9 (2)
C2—C3—C4—Zr1	70.4 (2)	C52—C51—C55—Zr3	−67.4 (2)
C3—C4—C5—C1	−2.5 (3)	C60—C56—C57—C58	1.9 (3)
Zr1—C4—C5—C1	−67.0 (2)	Zr3—C56—C57—C58	−67.75 (19)
C3—C4—C5—Zr1	64.4 (2)	C60—C56—C57—Zr3	69.68 (19)
C2—C1—C5—C4	−0.7 (3)	C56—C57—C58—C59	0.4 (3)
Zr1—C1—C5—C4	68.1 (2)	Zr3—C57—C58—C59	−66.3 (2)
C2—C1—C5—Zr1	−68.7 (2)	C56—C57—C58—Zr3	66.69 (18)
C10—C6—C7—C8	3.0 (3)	C57—C58—C59—C60	−2.6 (3)
Zr1—C6—C7—C8	−65.35 (19)	Zr3—C58—C59—C60	−69.2 (2)
C10—C6—C7—Zr1	68.39 (18)	C57—C58—C59—Zr3	66.6 (2)
C6—C7—C8—C9	−2.1 (3)	C58—C59—C60—C56	3.8 (3)
Zr1—C7—C8—C9	−67.22 (19)	Zr3—C59—C60—C56	−65.3 (2)
C6—C7—C8—Zr1	65.09 (19)	C58—C59—C60—Zr3	69.1 (2)
C7—C8—C9—C10	0.4 (3)	C57—C56—C60—C59	−3.5 (3)
Zr1—C8—C9—C10	−68.36 (19)	Zr3—C56—C60—C59	65.6 (2)
C7—C8—C9—Zr1	68.7 (2)	C57—C56—C60—Zr3	−69.17 (18)
C8—C9—C10—C6	1.5 (3)	C65—C61—C62—C63	5.2 (3)
Zr1—C9—C10—C6	−66.8 (2)	C66—C61—C62—C63	−142.3 (3)
C8—C9—C10—Zr1	68.37 (19)	Zr3—C61—C62—C63	−68.98 (19)
C7—C6—C10—C9	−2.8 (3)	C65—C61—C62—Zr3	74.18 (16)
Zr1—C6—C10—C9	66.66 (19)	C66—C61—C62—Zr3	−73.3 (2)
C7—C6—C10—Zr1	−69.49 (19)	C61—C62—C63—C64	−3.0 (3)
C15—C11—C12—C13	5.4 (3)	Zr3—C62—C63—C64	−63.5 (2)
C16—C11—C12—C13	−142.9 (3)	C61—C62—C63—Zr3	60.50 (17)
Zr1—C11—C12—C13	−69.05 (17)	C62—C63—C64—C65	−0.6 (3)
C15—C11—C12—Zr1	74.44 (16)	Zr3—C63—C64—C65	−60.95 (19)
C16—C11—C12—Zr1	−73.8 (2)	C62—C63—C64—Zr3	60.38 (19)
C11—C12—C13—C14	−3.4 (3)	C63—C64—C65—C61	3.9 (3)
Zr1—C12—C13—C14	−64.32 (18)	Zr3—C64—C65—C61	−61.37 (17)
C11—C12—C13—Zr1	60.92 (16)	C63—C64—C65—Zr3	65.3 (2)
C12—C13—C14—C15	−0.1 (3)	C62—C61—C65—C64	−5.6 (3)
Zr1—C13—C14—C15	−60.85 (17)	C66—C61—C65—C64	142.2 (3)
C12—C13—C14—Zr1	60.76 (17)	Zr3—C61—C65—C64	69.4 (2)
C13—C14—C15—C11	3.5 (3)	C62—C61—C65—Zr3	−74.98 (16)
Zr1—C14—C15—C11	−61.09 (16)	C66—C61—C65—Zr3	72.8 (2)
C13—C14—C15—Zr1	64.62 (17)	C62—C61—C66—C71	−39.4 (4)
C12—C11—C15—C14	−5.5 (3)	C65—C61—C66—C71	178.7 (2)
C16—C11—C15—C14	144.0 (2)	Zr3—C61—C66—C71	−110.9 (2)
Zr1—C11—C15—C14	69.13 (18)	C62—C61—C66—C67	−173.9 (2)
C12—C11—C15—Zr1	−74.59 (16)	C65—C61—C66—C67	44.2 (4)
C16—C11—C15—Zr1	74.8 (2)	Zr3—C61—C66—C67	114.6 (2)
C15—C11—C16—C17	43.8 (4)	C62—C61—C66—Zr3	71.5 (2)
C12—C11—C16—C17	−172.9 (2)	C65—C61—C66—Zr3	−70.4 (2)
Zr1—C11—C16—C17	115.8 (2)	C61—C66—C67—C68	−164.5 (3)
C15—C11—C16—C21	178.4 (2)	C71—C66—C67—C68	57.4 (3)
C12—C11—C16—C21	−38.3 (4)	Zr3—C66—C67—C68	−89.6 (3)
Zr1—C11—C16—C21	−109.6 (2)	C61—C66—C67—C72	76.2 (3)
C15—C11—C16—Zr1	−72.1 (2)	C71—C66—C67—C72	−62.0 (3)
C12—C11—C16—Zr1	71.3 (2)	Zr3—C66—C67—C72	151.0 (2)
C11—C16—C17—C18	−164.1 (3)	C66—C67—C68—C69	−58.8 (3)
C21—C16—C17—C18	57.3 (3)	C72—C67—C68—C69	60.4 (4)
Zr1—C16—C17—C18	−90.2 (3)	C67—C68—C69—C70	59.2 (4)
C11—C16—C17—C22	76.6 (3)	C67—C68—C69—C73	−60.5 (4)
C21—C16—C17—C22	−61.9 (3)	C68—C69—C70—C71	−59.6 (4)
Zr1—C16—C17—C22	150.59 (18)	C73—C69—C70—C71	60.1 (4)
C16—C17—C18—C19	−60.0 (3)	C61—C66—C71—C70	165.1 (2)
C22—C17—C18—C19	59.0 (3)	C67—C66—C71—C70	−57.2 (3)
C17—C18—C19—C23	−59.6 (4)	Zr3—C66—C71—C70	90.9 (3)
C17—C18—C19—C20	60.3 (3)	C61—C66—C71—C74	−76.0 (3)
C23—C19—C20—C21	59.9 (3)	C67—C66—C71—C74	61.7 (3)
C18—C19—C20—C21	−60.4 (4)	Zr3—C66—C71—C74	−150.2 (2)
C11—C16—C21—C20	165.5 (2)	C69—C70—C71—C66	59.2 (3)
C17—C16—C21—C20	−56.9 (3)	C69—C70—C71—C74	−59.7 (4)
Zr1—C16—C21—C20	92.4 (3)	C66—C67—C72—C75	60.9 (4)
C11—C16—C21—C24	−76.0 (3)	C68—C67—C72—C75	−59.9 (4)
C17—C16—C21—C24	61.7 (3)	C68—C69—C73—C75	60.1 (4)
Zr1—C16—C21—C24	−149.0 (2)	C70—C69—C73—C75	−59.5 (4)
C19—C20—C21—C16	59.3 (3)	C66—C71—C74—C75	−60.9 (4)
C19—C20—C21—C24	−59.7 (3)	C70—C71—C74—C75	59.1 (4)
C16—C17—C22—C25	60.6 (3)	C69—C73—C75—C74	59.6 (4)
C18—C17—C22—C25	−60.2 (3)	C69—C73—C75—C72	−60.1 (4)
C18—C19—C23—C25	59.9 (4)	C71—C74—C75—C73	−59.9 (4)
C20—C19—C23—C25	−59.1 (3)	C71—C74—C75—C72	60.1 (4)
C16—C21—C24—C25	−59.8 (3)	C67—C72—C75—C73	60.3 (4)
C20—C21—C24—C25	60.3 (3)	C67—C72—C75—C74	−60.1 (4)
C21—C24—C25—C23	−60.8 (3)	C80—C76—C77—C78	3.1 (3)
C21—C24—C25—C22	58.6 (3)	Zr4—C76—C77—C78	−65.7 (2)
C19—C23—C25—C24	59.9 (4)	C80—C76—C77—Zr4	68.8 (2)
C19—C23—C25—C22	−60.0 (4)	C76—C77—C78—C79	−3.3 (3)
C17—C22—C25—C24	−59.2 (3)	Zr4—C77—C78—C79	−69.4 (2)
C17—C22—C25—C23	60.7 (3)	C76—C77—C78—Zr4	66.1 (2)
C30—C26—C27—C28	3.0 (3)	C77—C78—C79—C80	2.4 (3)
Zr2—C26—C27—C28	−65.75 (19)	Zr4—C78—C79—C80	−67.0 (2)
C30—C26—C27—Zr2	68.7 (2)	C77—C78—C79—Zr4	69.4 (2)
C26—C27—C28—C29	−4.1 (3)	C78—C79—C80—C76	−0.5 (3)
Zr2—C27—C28—C29	−70.00 (19)	Zr4—C79—C80—C76	−66.35 (19)
C26—C27—C28—Zr2	65.91 (19)	C78—C79—C80—Zr4	65.88 (19)
C27—C28—C29—C30	3.7 (3)	C77—C76—C80—C79	−1.6 (3)
Zr2—C28—C29—C30	−66.1 (2)	Zr4—C76—C80—C79	67.0 (2)
C27—C28—C29—Zr2	69.80 (19)	C77—C76—C80—Zr4	−68.6 (2)
C28—C29—C30—C26	−1.9 (3)	C85—C81—C82—C83	3.6 (3)
Zr2—C29—C30—C26	−67.5 (2)	Zr4—C81—C82—C83	−64.6 (2)
C28—C29—C30—Zr2	65.6 (2)	C85—C81—C82—Zr4	68.1 (2)
C27—C26—C30—C29	−0.7 (3)	C81—C82—C83—C84	−4.8 (3)
Zr2—C26—C30—C29	68.6 (2)	Zr4—C82—C83—C84	−70.89 (19)
C27—C26—C30—Zr2	−69.24 (19)	C81—C82—C83—Zr4	66.1 (2)
C35—C31—C32—C33	3.2 (4)	C82—C83—C84—C85	4.2 (3)
Zr2—C31—C32—C33	−65.4 (3)	Zr4—C83—C84—C85	−66.8 (2)
C35—C31—C32—Zr2	68.6 (2)	C82—C83—C84—Zr4	70.98 (19)
C31—C32—C33—C34	−1.6 (4)	C83—C84—C85—C81	−2.0 (3)
Zr2—C32—C33—C34	−67.2 (3)	Zr4—C84—C85—C81	−67.0 (2)
C31—C32—C33—Zr2	65.7 (2)	C83—C84—C85—Zr4	64.9 (2)
C32—C33—C34—C35	−0.7 (4)	C82—C81—C85—C84	−1.0 (4)
Zr2—C33—C34—C35	−70.1 (2)	Zr4—C81—C85—C84	67.3 (2)
C32—C33—C34—Zr2	69.4 (3)	C82—C81—C85—Zr4	−68.2 (2)
C33—C34—C35—C31	2.7 (4)	C90—C86—C87—C88	5.4 (3)
Zr2—C34—C35—C31	−66.9 (2)	C91—C86—C87—C88	−142.9 (3)
C33—C34—C35—Zr2	69.6 (3)	Zr4—C86—C87—C88	−70.01 (19)
C32—C31—C35—C34	−3.7 (4)	C90—C86—C87—Zr4	75.38 (16)
Zr2—C31—C35—C34	65.8 (2)	C91—C86—C87—Zr4	−72.9 (2)
C32—C31—C35—Zr2	−69.4 (2)	C86—C87—C88—C89	−3.7 (3)
C41—C36—C37—C38	−143.3 (2)	Zr4—C87—C88—C89	−65.40 (19)
C40—C36—C37—C38	5.1 (3)	C86—C87—C88—Zr4	61.68 (16)
Zr2—C36—C37—C38	−68.67 (19)	C87—C88—C89—C90	0.5 (3)
C41—C36—C37—Zr2	−74.6 (2)	Zr4—C88—C89—C90	−60.19 (18)
C40—C36—C37—Zr2	73.81 (16)	C87—C88—C89—Zr4	60.69 (18)
C36—C37—C38—C39	−3.0 (3)	C88—C89—C90—C86	2.9 (3)
Zr2—C37—C38—C39	−63.50 (19)	Zr4—C89—C90—C86	−60.38 (16)
C36—C37—C38—Zr2	60.52 (17)	C88—C89—C90—Zr4	63.30 (19)
C37—C38—C39—C40	−0.5 (3)	C87—C86—C90—C89	−5.0 (3)
Zr2—C38—C39—C40	−61.15 (18)	C91—C86—C90—C89	142.7 (3)
C37—C38—C39—Zr2	60.66 (18)	Zr4—C86—C90—C89	69.21 (19)
C38—C39—C40—C36	3.8 (3)	C87—C86—C90—Zr4	−74.26 (16)
Zr2—C39—C40—C36	−61.64 (17)	C91—C86—C90—Zr4	73.5 (2)
C38—C39—C40—Zr2	65.41 (19)	C90—C86—C91—C96	171.3 (2)
C37—C36—C40—C39	−5.4 (3)	C87—C86—C91—C96	−46.2 (4)
C41—C36—C40—C39	143.7 (2)	Zr4—C86—C91—C96	−116.8 (2)
Zr2—C36—C40—C39	69.33 (18)	C90—C86—C91—C92	37.3 (4)
C37—C36—C40—Zr2	−74.77 (17)	C87—C86—C91—C92	179.8 (2)
C41—C36—C40—Zr2	74.4 (2)	Zr4—C86—C91—C92	109.2 (2)
C37—C36—C41—C46	−39.3 (4)	C90—C86—C91—Zr4	−71.9 (2)
C40—C36—C41—C46	177.4 (2)	C87—C86—C91—Zr4	70.6 (2)
Zr2—C36—C41—C46	−111.5 (2)	C86—C91—C92—C93	−166.3 (3)
C37—C36—C41—C42	−173.9 (2)	C96—C91—C92—C93	56.8 (3)
C40—C36—C41—C42	42.8 (3)	Zr4—C91—C92—C93	−92.6 (3)
Zr2—C36—C41—C42	113.9 (2)	C86—C91—C92—C97	75.3 (3)
C37—C36—C41—Zr2	72.2 (2)	C96—C91—C92—C97	−61.6 (3)
C40—C36—C41—Zr2	−71.2 (2)	Zr4—C91—C92—C97	149.0 (2)
C36—C41—C42—C43	−164.6 (2)	C91—C92—C93—C94	−59.1 (3)
C46—C41—C42—C43	57.1 (3)	C97—C92—C93—C94	59.6 (4)
Zr2—C41—C42—C43	−91.2 (3)	C92—C93—C94—C95	59.8 (3)
C36—C41—C42—C47	76.3 (3)	C92—C93—C94—C98	−60.0 (4)
C46—C41—C42—C47	−62.0 (3)	C93—C94—C95—C96	−59.5 (4)
Zr2—C41—C42—C47	149.7 (2)	C98—C94—C95—C96	60.2 (4)
C41—C42—C43—C44	−59.4 (3)	C94—C95—C96—C91	59.2 (4)
C47—C42—C43—C44	59.9 (3)	C94—C95—C96—C99	−59.8 (4)
C42—C43—C44—C48	−60.1 (3)	C86—C91—C96—C95	164.8 (3)
C42—C43—C44—C45	60.1 (4)	C92—C91—C96—C95	−57.3 (3)
C48—C44—C45—C46	60.4 (3)	Zr4—C91—C96—C95	89.9 (3)
C43—C44—C45—C46	−60.2 (3)	C86—C91—C96—C99	−75.9 (3)
C36—C41—C46—C45	165.6 (2)	C92—C91—C96—C99	62.0 (3)
C42—C41—C46—C45	−56.9 (3)	Zr4—C91—C96—C99	−150.8 (2)
Zr2—C41—C46—C45	92.1 (3)	C91—C92—C97—C100	60.0 (4)
C36—C41—C46—C49	−75.6 (3)	C93—C92—C97—C100	−59.6 (4)
C42—C41—C46—C49	61.9 (3)	C95—C94—C98—C100	−59.8 (5)
Zr2—C41—C46—C49	−149.14 (18)	C93—C94—C98—C100	59.4 (4)
C44—C45—C46—C41	59.1 (3)	C95—C96—C99—C100	59.9 (4)
C44—C45—C46—C49	−59.7 (3)	C91—C96—C99—C100	−60.8 (4)
C43—C42—C47—C50	−60.4 (3)	C94—C98—C100—C97	−60.1 (4)
C41—C42—C47—C50	60.3 (3)	C94—C98—C100—C99	60.0 (4)
C43—C44—C48—C50	59.3 (3)	C92—C97—C100—C98	60.8 (4)
C45—C44—C48—C50	−60.3 (4)	C92—C97—C100—C99	−59.3 (3)
C41—C46—C49—C50	−60.3 (3)	C96—C99—C100—C98	−60.5 (4)
C45—C46—C49—C50	59.9 (3)	C96—C99—C100—C97	59.8 (4)
C44—C48—C50—C47	−59.3 (3)	C106—C101—C102—C103	6.1 (14)
C44—C48—C50—C49	60.5 (3)	C107—C101—C102—C103	−177.2 (13)
C42—C47—C50—C48	60.4 (3)	C101—C102—C103—C104	−6.5 (15)
C42—C47—C50—C49	−59.2 (3)	C102—C103—C104—C105	2.5 (17)
C46—C49—C50—C48	−60.6 (3)	C103—C104—C105—C106	2.4 (18)
C46—C49—C50—C47	59.2 (4)	C104—C105—C106—C101	−2.8 (17)
C55—C51—C52—C53	2.5 (4)	C107—C101—C106—C105	−178.5 (13)
Zr3—C51—C52—C53	−65.5 (2)	C102—C101—C106—C105	−1.4 (16)
C55—C51—C52—Zr3	68.0 (2)	C108—C109—C110—C111	86.9 (18)
C51—C52—C53—C54	−4.6 (3)	C109—C110—C111—C112	−165.7 (13)
Zr3—C52—C53—C54	−72.0 (2)	C110—C111—C112—C113	178.6 (14)
C51—C52—C53—Zr3	67.4 (2)		
